# Cell-composition effects in the analysis of DNA methylation array data: a mathematical perspective

**DOI:** 10.1186/s12859-015-0527-y

**Published:** 2015-03-21

**Authors:** E Andres Houseman, Karl T Kelsey, John K Wiencke, Carmen J Marsit

**Affiliations:** 1 School of Biological and Population Health Sciences, College of Public Health and Human Sciences, Oregon State University, Corvallis, OR USA; 2 Department of Epidemiology, Brown University School of Public Health, Providence, RI USA; 3 Departments of Neurological Surgery, and Division of Epidemiology, University of California San Francisco, San Francisco, CA USA; 4 Department of Community and Family Medicine, Dartmouth Medical School, Hanover, NH USA

**Keywords:** Epigenetics, Epigenome-wide-association, Epigenomics, Immune, Infinium

## Abstract

**Background:**

The impact of cell-composition effects in analysis of DNA methylation data is now widely appreciated. With the availability of a reference data set consisting of DNA methylation measurements on isolated cell types, it is possible to impute cell proportions and adjust for them, but there is increasing interest in methods that adjust for cell composition effects when reference sets are incomplete or unavailable.

**Results:**

In this article we present a theoretical basis for one such method, showing that the total effect of a phenotype on DNA methylation can be decomposed into orthogonal components, one representing the effect of phenotype on proportions of major cell types, the other representing either subtle effects in composition or global effects at focused loci, and that it is possible to separate these two types of effects in a finite data set. We demonstrate this principle empirically on nine DNA methylation data sets, showing that the first few principal components generally contain a majority of the information on cell-type present in the data, but that later principal components nevertheless contain information about a small number of loci that may represent more focused associations. We also present a new method for determining the number of linear terms to interpret as cell-mixture effects and demonstrate robustness to the choice of this parameter.

**Conclusions:**

Taken together, our work demonstrates that reference-free algorithms for cell-mixture adjustment can produce biologically valid results, separating cell-mediated epigenetic effects (i.e. apparent effects arising from differences in cell composition) from those that are not cell mediated, and that in general the interpretation of associations evident from DNA methylation should be carefully considered.

**Electronic supplementary material:**

The online version of this article (doi:10.1186/s12859-015-0527-y) contains supplementary material, which is available to authorized users.

## Background

In the last decade, numerous publications have reported associations between DNA methylation profiles in a single tissue (the majority of studies published to date interrogate peripheral blood leukocyte mixtures) and disease states or exposure phenotypes. For example, DNA methylation profiles measured in blood have been shown to correlate with ovarian cancer [1], bladder cancer [2], cardiovascular disease [3], obesity [4], and environmental exposures [5-7]. These associations have led to an interest in *epigenome-wide association studies* (EWAS), which aim to investigate associations between DNA methylation and health or exposure phenotypes across the genome. DNA methylation and coordinated chromatin alterations are partially responsible for coordination of gene expression in individual cells [8-10]. Consequently, normal tissue development, individual cellular differentiation and cellular lineage determination are regulated by epigenetic mechanisms [9]. This necessarily means that DNA methylation shows substantial variation across tissue types [11] as well as individual cell types, demonstrated particularly clearly amongst the distinct types of leukocytes [8]. This biological directive leads to significant and potentially underappreciated problems in interpreting the results of EWAS studies, which have predominantly utilized peripheral blood samples (whole blood or buffy coat) as the source of DNA for these analyses. There is a profound and fundamental difference between “environmentally induced DNA methylation” and environmentally induced (or, more properly, “biologically selected”) changes in the underlying landscape of cellular subtypes within a given sample. These involve completely distinct events - the former an intra-nuclear enzymatic action perhaps mediated at the level of the cell and the latter a signaling event that likely involves the entire immune cascade. There is limited evidence for the existence of the former phenomenon, e.g. numerous reports that collectively demonstrate a dose-related effect on DNA methylation along the AHRR gene in distinctly different tissues [7,12,13]. However, the second mechanism may predominate in most applications of EWAS, as has been demonstrated in a number of recent papers [14-17]. In cases where potentially both phenomena are occurring, the phenotypic effect on the distribution of cell types within a sampled tissue, i.e. *cell-composition effects*, represents a confounder of associations driven by the intra-nuclear activity.

Several algorithms have been proposed to address this issue in a mathematically simple manner. Houseman et al. (2012) proposed a procedure for imputing a restricted set of cell type proportions directly from DNA methylation data; a limitation of this approach is that only cell types for which a *reference data set* exists can be evaluated, i.e. an assembly of DNA methylation measurements on isolated cell types [18]. Liu et al. (2013) demonstrated the use of these proportions as adjustment covariates in an EWAS [17]. Subsequently, we recently proposed a *reference-free* algorithm that potentially obviates this problem, as it can be used in studies where no reference data set exists [19]; in the paper we validated the method against the reference-based approach, and this algorithm has already been applied successfully, e.g. to discover a DNA methylation biomarker for Wilms tumor [20]. Several similar algorithms have also recently been published, including a method specific to brain tissue [21] as well as the *EWASher* method [22], which is similar in spirit to our algorithm but reverses the role of dependent and independent variables. The reference-free algorithm employs a singular value decomposition (SVD) to separate cell-composition effect from a direct effect (generally interpreted as the intra-nuclear activity). Thus, the algorithm entails very strong linearity assumptions, i.e. that the linear structures underlying variability in a data set will necessarily correspond to mixing of DNA methylation via cell composition. This is true also of *EWASher*, as well as the *surrogate variable analysis* (SVA) and *independent SVA* (ISVA) algorithms [23,24] that are similar to our proposed method (see below) and have previously been used to account both for technical artifacts and cell mixture effects. The goal of the present article is to justify such linearity assumptions from a rigorous biological and mathematical framework, demonstrating that the linear space of epigenetic effects can be partitioned into two subspaces, one representing cell-mediated effects (i.e. those arising from differences in cell composition) and the other representing the remaining, non-cell-mediated effects. Projection of total epigenetic effect onto the latter space thus recovers the non-cell-mediated associations and our present work thus justifies the application of our previously published algorithm, as well as similar algorithms that rely on similar linear decompositions.

In general, reference-based approaches will be superior to reference-free approaches because the deconvolution required for estimating cell type proportions is essentially supervised by basis vectors that have direct biological interpretations. In the reference-based procedure, cell proportions are obtained by projecting whole-tissue DNA methylation data onto linear spaces spanned by cell-type-specific methylation profiles for a specific set of cyotosine-phosphate-guanine dinucleotides (CpGs) that distinguish the cell types, so-called differentially methylated regions (DMRs). However, we envision that epigenetic approaches will be applied in the future to tissues of diverse cell origin that contain unique combinations of cell type DMRs. In many of these cases, prior knowledge of major constituent cell types (their DMRs and their DNA methylation profiles) may be lacking. In such cases, the reference-free approach is readily applicable. However, even the complexity of a given sample may be unknown; even in blood, activation of distinct types of cells may play an important role in some diseases (e.g. [25]), in which case results of reference-free approaches could in principle depend upon the assumed level of complexity. Thus, critical to the algorithm is its sensitivity of results to choice of a parameter that represents the complexity, the dimension *k* of the latent linear association driven by cell composition, widely (though perhaps incorrectly) interpreted as the number of cell types. As we demonstrate below, choice of this parameter somewhat impacts the results of the algorithm. Consequently, as a second goal of this article, we also examine in detail the choice of dimension, its potential biological significance, and its impact on data analysis. In particular, we argue that, mathematically, major cell-composition effects can be distinguished from other effects by a choice of two orthogonal vector spaces, and consequently the SVD of a certain matrix provides information about cellular type. By associating the largest singular values of this decomposition with the space corresponding to major cell types, it becomes possible to distinguish cell-composition effects from other effects. The choice of the number *k* of singular values to associate with cell-composition effects then drives the analytical results. Over a range of values for *k*, we apply this analysis to nine distinct data sets, showing that in most cases, results will remain stable for a wide range of choices of *k*. We also show that the original method proposed for selecting *k* may not always reliably find the stable range and propose a simple alternative procedure that is slightly more reliable. Finally, we discuss the implications of these results to biological interpretation of EWAS.

## Results and discussion

Cell-composition effects represent a potential mediator of associations observed between a phenotype (disease state or environmental exposure) and DNA methylation measured in a heterogeneous tissue, as well as a confound of “direct” associations (presumed to represent intra-nuclear activity or “direct” action of the exposure, producing DNA methylation without disturbing the cellular distribution). Under reasonable regression assumptions (no effect modification by cell composition and independence of cell composition with the errors in measurement of DNA methylation), several techniques are currently available for analyzing DNA methylation data while accounting for cellular heterogeneity. All of them assume essentially the following linear model for *m* CpG loci measured on *n* subjects:

1 $$ \mathbf{Y}=\mathbf{B}{\mathbf{X}}^T+\mathbf{M}{\boldsymbol{\Omega}}^T+\mathbf{E}, $$

where **Y** is an *m* × *n* matrix of average beta values, **X** is an *n* × *d* design matrix of phenotype variables and potential confounders (for a total of *d* covariates including an intercept), **B** is the *m* × *d* matrix of regression coefficients representing direct effects, **MΩ** ^*T*^ represents a linear mixture effect, with **M** an *m* × *k*′ matrix representing *m* CpG-specific methylation states for *k*′ cell types, **Ω** is an *n* × *k*′ matrix representing subject-specific cell-type distributions (each row representing the cell-type proportions for a given subject), and **E** is an *m* × *n* matrix of errors with *E*(**E**) = **0**_*m* × *n*_. We discuss the meaning of “direct effect” below. Note that the value *k*′ is assumed to be known in advance, although we have earlier proposed estimating it by an application of random matrix theory originally described by Teschendorff et al. (2011) [24]. Note also that the entries of **Y**, of **M**, and of **Ω** are assumed to lie in the unit interval, and that the rows of **Ω** sum to one. Finally, note that (1) is true even when **M**, with a very large value of *k*′, exhaustively characterizes all possible types of cells in the target tissue, although with finite data set it may not be possible to estimate certain parameters in (1).

### Linear characterization of cell mixture

Consider a particular cell type *T*, which may be as general as a leukocyte or a lymphocyte, or as specific as a CD4+ T lymphocyte or CD4+ regulatory T cell (T_reg_). We assume *T* has methylation profile **μ** = (*μ*_1_, …, *μ*_*n*_)^*T*^ representing the mean methylation state for that type, where the implied expectation averages the methylation states of all subtypes with probability weights equal to the mean distribution of the subtypes in a target human population. As a concrete example, let us suppose *T* represents all CD4+ T lymphocytes. If now two subtly different subtypes (e.g. T_reg_ cells and helper T_H_ cells) are defined by differences in the epigenetic states of only the first r loci, e.g. between $\left({\mu}_{10}^{*},\dots, {\mu}_{r0}^{*}\right)$ and $\left({\mu}_{11}^{*},\dots, {\mu}_{r1}^{*}\right),$ and each of these types occurs in population average proportions *ω*_0_ and *ω*_1_ respectively (with *ω*_0_ + *ω*_1_ = 1), then the DNA methylation states of the two subtypes are respectively ${\boldsymbol{\upmu}}_0={\left({\mu}_{10}^{*},\dots, {\mu}_{r0}^{*},{\mu}_{r+1},\dots, {\mu}_n\right)}^T$ and ${\boldsymbol{\upmu}}_1={\left({\mu}_{11}^{*},\dots, {\mu}_{r1}^{*},{\mu}_{r+1},\dots, {\mu}_n\right)}^T,$ with **μ** = **μ**_0_ *ω*_0_ + **μ**_1_ *ω*_1_. Note that this is true even for a very general type *T* such as all leukocytes, which may be conceived as a mixture of myeloid and lymphoid cell lineages. It will also be true of solid tissues; for example, even adipose tissue has been described to have multiple different types of adipocytes [26].

This concept is related to the idea of a recursive partitioning mixture model, which has been used previously in the analysis of DNA methylation data [27]. Commonly, normal constituent cell types of a complex tissue will have different functions, each represented by distinct epigenetic states, so that the cell types can be partitioned from “coarse” to “fine” by recursively partitioning each constituent type in the manner just described. However, in cases where a single alteration is observed across many types or even tissues (e.g. smoking-related effects on the AHRR gene locus) it is possible to mathematically represent such an alteration as a shift in cell type, simply by taking *r* = 1 (or a small number) in the above partitioning analysis. For the moment we ignore the biological origin of such an alteration (wide-scale and dose-dependent intra-nuclear action or signaling cascade and cell selection), noting that the mathematical representation is identical.

It follows from elementary linear algebra that *n* arbitrary cell types could be defined for an array with *n* loci (although likely many fewer would have biological meaning, i.e. correspond to true biological function). In other words, it is possible to define a linearly independent set of *n* cell-type state profiles {**μ**_1_, **μ**_2_, …, **μ**_*n*_}. Theoretically it is also possible to define the set of *n* profiles with state vectors that are orthogonal to each other, although again it is extremely unlikely that such an orthogonal set of profiles will correspond to biologically and functionally meaningful cell types. For example, consider two gross subtypes **μ**_0_ = (0, *μ*_2_, …, *μ*_*n*_)^*T*^ and **μ**_1_ = (1, *μ*_2_, …, *μ*_*n*_)^*T*^, differing in methylation state only at the first locus. Each of these states may be highly meaningful in a biological sense, but they are clearly not orthogonal for general values of 0 ≤ *μ* ≤ 1. To orthogonalize the set {**μ**_0_, **μ**_1_}, we must instead consider a decomposition such as {**μ**_0_, **ε**_1_}, where **ε**_1_ = (1, 0, …, 0)^*T*^ is a unit vector corresponding to (presumably) no biologically functional cell type. Thus, both profiles **μ**_0_ and **μ**_1_ may be obtained as linear combinations of **μ**_0_ and **ε**_1_, but profile **ε**_1_ likely does not correspond to any biological function. Nevertheless, if we are concerned with a small relevant subset of *k*′ major subtypes $\left\{{\boldsymbol{\upmu}}_1,{\boldsymbol{\upmu}}_2,\dots, {\boldsymbol{\upmu}}_{k^{\prime }}\right\},$ it is possible to differentiate these types from more subtle forms of variation by decomposing the space spanned by {**μ**_1_, **μ**_2_, …, **μ**_*n*_} into a vector subspace $\mathsf{M}= span\left\{{\boldsymbol{\upmu}}_1,{\boldsymbol{\upmu}}_2,\dots, {\boldsymbol{\upmu}}_{k^{\prime }}\right\}$ and its complementary vector space ${\mathsf{M}}^{\perp }$, orthogonal to M. This is achieved by partitioning each major type into its constituent types in the manner described above, i.e. determining *r* loci that distinguish the subtypes from a parent type, and subsequently using a Gram-Schmidt orthonormalization procedure to orthogonalize the vector subspace corresponding to those loci. For example, consider type *T* and the sub-vector ${\boldsymbol{\upmu}}^{*}={\left({\mu}_1^{*},\dots, {\mu}_r^{*}\right)}^T$ of mean states for the loci that distinguish the subtypes of *T*, and let $\left\{{\boldsymbol{\upvarepsilon}}_1^{*},\dots, {\boldsymbol{\upvarepsilon}}_r^{*}\right\}$ be a corresponding set of *r* unit vectors. Starting with μ*, *r* - 1 additional vectors can be chosen from $span\left\{{\boldsymbol{\upvarepsilon}}_1^{*},\dots, {\boldsymbol{\upvarepsilon}}_r^{*}\right\}$ to form an orthogonal basis for $span\left\{{\boldsymbol{\upmu}}^{*},{\boldsymbol{\upvarepsilon}}_1^{*},\dots, {\boldsymbol{\upvarepsilon}}_r^{*}\right\},$, and these vectors define members of ${\mathsf{M}}^{\perp }$ that together with $\boldsymbol{\upmu} \in \mathsf{M}$ identify all subtypes of *T*. Thus, in general, matrix **M** in equation (1) may be augmented as $\tilde{\mathbf{M}}=\left[{\mathbf{M}}^{(bio)},{\mathbf{M}}^{\left(\perp \right)}\right]$ to exhaustively characterize all types that can be defined within $\tilde{\mathsf{M}}=\mathsf{M}\oplus {\mathsf{M}}^{\perp },$ where the column vectors of **M** ^(*bio*)^ are orthogonal to the column vectors in **M** ^(⊥)^ and correspond to epigenetic states that are biologically relevant to major cell types. In particular, the column vectors of **M** ^(⊥)^ form a linear basis for the effects that are not mediated by cell type. We remark that $\tilde{\mathsf{M}}$ is a vector space of dimension *n*, although only a convex subset of $\tilde{\mathsf{M}}$ satisfies constraints on the values that mean methylation profiles can take, and a much smaller subset corresponds to biologically meaningful profiles.

### Mediation by phenotypic cell composition effects

In addition to the linear model (1) defining mixtures of DNA methylation states, we assume that the matrix of cell-type proportions **Ω** in (1) is a random variable potentially associated with **X**. Although a Dirichlet model would most appropriately model the rows of **Ω**, a reasonable and computationally efficient linear approximation is as follows:

2 $$ \boldsymbol{\Omega} =\mathbf{X}\boldsymbol{\Gamma } +\boldsymbol{\Xi}, $$

where **Γ** is a *d* × *k*′ matrix of covariate effects upon cell proportion and **Ξ** is an *n* × *k*′ error matrix. Note that equation (1) explicitly omits interaction between **X** and **Ω**, which is likely adequate for most problems. With the additional assumption that **E** and **Ξ** are independent (and independent of **X**), and substituting (2) in (1), we have

3 $$ \mathbf{Y}=\mathbf{B}{\mathbf{X}}^T+\mathbf{M}{\boldsymbol{\Omega}}^T+\mathbf{E}=\left(\mathbf{B}+\mathbf{M}{\boldsymbol{\Gamma}}^T\right){\mathbf{X}}^T+\left(\mathbf{M}\boldsymbol{\Xi } +\mathbf{E}\right). $$

The total effect of **X** upon **Y** is *E*(**Y**|**X**) = (**B** + **MΓ** ^*T*^)**X** ^*T*^, the direct effect is **BX** ^*T*^, and the mediated, or *cell-composition effect*, is **ΔX** ^*T*^, where **Δ** = **MΓ** ^*T*^. Note that (3) can be written as **Y** = **AX** ^*T*^ + **R**,, where **A** = **MΓ** ^*T*^ + **B** and the error matrix **R** = **MΞ** + **E** includes a term that depends on the cell-type-specific coefficient matrix **M**. Here, **A** is the *total* effect of phenotype matrix **X**. Following from the previous section, we replace **M** in (1) with **M** ^(*bio*)^, assuming all cell types that mediate phenotypic effects are captured in **M** ^(*bio*)^, and explore the relationship of (1) to the *m* × (*n* − *k*′) matrix **M** ^(⊥)^. Note that two types of “direct” effects (understood from a molecular point of view) are possible. The first is a subtle alteration of a major subtype. Adopting the notation of the previous section and without loss of generality, assume that exactly one locus characterizes such an alteration (from ${\mu}_0^{*}$ to ${\mu}_1^{*}$), **μ** is the average profile across altered and unaltered types, and that **ε** is a unit vector nonzero for every locus except the altered one. If *ω*_0_ is the mean proportion of unaltered cells (of the given type) and *ω*_1_ is the corresponding mean proportion of altered cells, *ω*_0_ + *ω*_1_ = 1, then ${\overline{\mu}}^{*}={\omega}_0{\mu}_0^{*}+{\omega}_1{\mu}_1^{*}$ is the average methylation at the altered locus, $\boldsymbol{\upmu} +\left({\mu}_0^{*}-{\overline{\mu}}^{*}\right)\boldsymbol{\upvarepsilon}$ is the unaltered profile, $\boldsymbol{\upmu} +\left({\mu}_1^{*}-{\overline{\mu}}^{*}\right)\boldsymbol{\upvarepsilon}$ is the altered profile, and a mean shift in distribution of types, (*Δω*_0_, *Δω*_1_), *Δω*_0_ + *Δω*_1_ = 0, is characterized by a mean methylation difference of $\Delta \boldsymbol{\upmu} =\Delta {\omega}_0\left[\boldsymbol{\upmu} +\left({\mu}_0^{*}-{\overline{\mu}}^{*}\right)\boldsymbol{\upvarepsilon} \right]+\Delta {\omega}_1\left[\boldsymbol{\upmu} +\left({\mu}_1^{*}-{\overline{\mu}}^{*}\right)\boldsymbol{\upvarepsilon} \right]=\left[\Delta {\omega}_0\left({\mu}_0^{*}-{\overline{\mu}}^{*}\right)+\Delta {\omega}_1\left({\mu}_1^{*}-{\overline{\mu}}^{*}\right)\right]\boldsymbol{\upvarepsilon}$.

In other words, the effect is captured entirely within the vector space ${\mathsf{M}}^{\perp }$. A second type of “direct” effect is a wholesale change across every cell type (e.g. carcinogenic transformation of a normal cell). In this scenario, every cell type characterized by M has the same alteration, in which case (by an argument similar to that above) the effect is also captured entirely within the space ${\mathsf{M}}^{\perp }$. In summary, a “direct” effect **BX** ^*T*^ will be any effect that lies within ${\mathsf{M}}^{\perp }$, outside the space spanned by the profiles of the major cell types of interest. Consequently, we have **B** = **M** ^(⊥)^ **Η** ^*T*^, expressed now as a mixture of subtler effects living in ${\mathsf{M}}^{\perp }$ with projection coefficient **H**, a *d* × (*n* − *k*′) matrix. This in turn implies **A** = **M** ^(*bio*)^ **Γ** ^*T*^ + **M** ^(⊥)^ **Η** ^*T*^. As in Houseman et al. (2014), we concatenate the total effects matrix **A** and the total error matrix **R**, decomposing as follows:

4 $$ \left[\begin{array}{cc}\hfill \mathbf{A}\hfill & \hfill \mathbf{R}\hfill \end{array}\right]=\left[\begin{array}{cc}\hfill {\mathbf{M}}^{(bio)}\hfill & \hfill {\mathbf{M}}^{\left(\perp \right)}\hfill \end{array}\right]{\left[\begin{array}{cc}\hfill \boldsymbol{\Gamma} \hfill & \hfill \mathbf{H} \hfill \\ {}\hfill \boldsymbol{\Xi} \hfill & \hfill {\mathbf{E}}^T\boldsymbol{\Pi} \hfill \end{array}\right]}^T+\left[\begin{array}{cc}\hfill \mathbf{O}\hfill & \hfill \mathbf{E}-{\mathbf{M}}^{\left(\perp \right)}{\boldsymbol{\Pi}}^T\mathbf{E}\hfill \end{array}\right] $$

where **Π** = **M** ^(⊥)^(**M** ^(⊥)*T*^ **M** ^(⊥)^)^− 1^ is the projection coefficient onto ${\mathsf{M}}^{\perp }$. In large samples, the error **E** should be asymptotically orthogonal to ${\mathsf{M}}^{\perp }$, therefore the second term in (4) is stochastically negligible. Note that if the singular values of the first term are non-degenerate, then the term has a unique SVD and, consequently $$ {\left[\begin{array}{cc}\hfill {\boldsymbol{\Gamma}}^T\hfill & \hfill {\boldsymbol{\Xi}}^T\hfill \end{array}\right]}^T $$ is orthogonal to $$ {\left[\begin{array}{cc}\hfill {\mathbf{H}}^T\hfill & \hfill {\boldsymbol{\Pi}}^T\mathbf{E}\hfill \end{array}\right]}^T $$. This motivates the use of the SVD of $$ \left[\begin{array}{cc}\hfill \mathbf{A}\hfill & \hfill \mathbf{R}\hfill \end{array}\right] $$ to recover M. Note that M could be recovered from **R** alone, a fact that underlies the application of *independent surrogate variable analysis* (ISVA, [24]) to adjust for cell composition effects. However, since **A** also contains information about M, it adds additional information to the decomposition. **A** alone cannot typically be used to recover M because *d* < *k*′ in most applications.

### Linear expansion of total effect

The concatenated matrix $$ \left[\begin{array}{cc}\hfill \mathbf{A}\hfill & \hfill \mathbf{R}\hfill \end{array}\right] $$ has singular value decomposition $$ \left[\begin{array}{cc}\hfill \mathbf{A}\hfill & \hfill \mathbf{R}\hfill \end{array}\right]=\mathbf{U}\;\boldsymbol{\Lambda}\;{\mathbf{V}}^T $$, where **U** = (**u**_1_, …, **u**_*n* + *d*_) is an orthogonal *m* × (*n* + *d*) matrix, **Λ** = *diag*(*λ*_1_, …, *λ*_*n* + *d*_) is a diagonal (*n* + *d*) × (*n* + *d*) matrix, and **V** = (**v**_1_, …, **v**_*n* + *d*_) is an orthogonal (*n* + *d*) × (*n* + *d*) matrix. Note that decomposition is algebraically equivalent to $$ \left[\begin{array}{cc}\hfill \mathbf{A}\hfill & \hfill \mathbf{R}\hfill \end{array}\right]={\displaystyle {\sum}_{j=1}^{n+d}{\lambda}_j{\mathbf{u}}_j{\mathbf{v}}_j^T}, $$ and therefore

5 $$ \mathbf{A}=\mathbf{U}\;\boldsymbol{\Lambda}\;{\mathbf{V}}^{*T}={\displaystyle {\sum}_{j=1}^{n+d}{\lambda}_j{\mathbf{Q}}_j}, $$

Where **V** ^*^ is the matrix consisting of the first *d rows* of **V**, ${\mathbf{Q}}_j={\mathbf{u}}_j{\mathbf{v}}_j^{*T}$, and ${\mathbf{v}}_j^{*}$ is the *j* ^th^ column of **V** ^*^. Thus the singular value decomposition effectively represents a linear expansion of **A** by *n* + *d* terms. Note that $A \in {R^{m \times d}}$, a vector space of dimension > *n + d*, so the expansion need not be overdetermined. If follows from (4) that the orthogonal columns of **U** are partitioned into two sets, those that span M and those that span ${\mathsf{M}}^{\perp }$. Thus, the terms **Q**_*j*_ contribute unambiguously either to cell-composition effects on the targeted cell types or else to direct effects lying outside the targeted types.

By convention, the singular values (diagonal elements of **Λ**) are ordered from greatest to least. Because we expect the variation in DNA methylation driven by differentiation of major cell types to dominate the variation among elements of $\tilde{\mathsf{M}}$, it follows that the largest singular values should correspond to basis vectors of M, and therefore we interpret the initial terms of (5) as cell-composition effects. However, it remains unclear how many initial terms *k* to select. Note that while we expect the value *k* to loosely correlate with the number of cell types *k*′, a direct correspondence may be difficult to establish in empirical data sets, as we demonstrate below. A reasonable approach is to vary *k* and examine the impact on estimates ${\boldsymbol{\Delta}}_k={\displaystyle {\sum}_{j=1}^k{\lambda}_j{\mathbf{Q}}_j}$ and ${\mathbf{B}}_k={\displaystyle {\sum}_{j=k+1}^{n+d}{\lambda}_j{\mathbf{Q}}_j}$ obtained by each choice of *k*.

### Results of empirical evaluation of theoretical concepts

Above we have argued that the total effect **A** of phenotype on DNA methylation can be decomposed into two orthogonal terms, **Δ**_*k*_, the effect of a phenotype on the distribution of major cell types, and **B**_*k*_, which represents the effect of the phenotype on subtle variants of one cell type or global effects (across cell type) focused at single loci. The dimension parameter *k* can be chosen by a method we have proposed previously [19,24] or by a new method we propose below in Methods, which seeks the value for which **Δ**_*k*_ is most “stable”, i.e. changes least across adjacent values of *k*. To evaluate the theory and demonstrate the phenomenon in finite data sets, we applied the proposed method to nine DNA methylation data sets obtained from Gene Expression Omnibus (GEO), each described in Table 1. Five data sets were generated from the HumanMethylation27 (27K) platform and four data sets from the HumanMethylation450 (450K) platform. Two are well-known reference data sets for blood, three were generated from epidemiology studies assaying whole/peripheral blood, one consists only of invasive breast tumors, and three others consist of comparisons of normal and pathological tissues (gastric, liver, and arterial).

**Table 1:** **Data sets analyzed**

	**GEO accession**	**Platf**	**Ref**	**Description**	**n**	**Covariate model**
Ref. Blood	GSE39981	27K	[18,52]	Human leukocyte subtypes purified from whole blood samples.	73	[**whole**|gran|mono|B|NK|CD4 + T|CD8 + T|Pan-T]
	GSE35069	450K	[47]	Human leukocyte subtypes purified from whole blood samples.	54	[**whole**|neut|eos|gran|mono|B|NK|CD4 + T|CD8 + T]
Blood	GSE30229*	27K	[16]	Peripheral blood from 92 head and neck squamous cell carcinoma (HNSCC) patients and 92 controls.	184	[**control**|HNSCC] + age
	GSE19711	27K	[1]	Whole blood from 131 ovarian cancer cases (drawn pre-treatment) and 274 controls.	405	[**control**|ov Cancer] + age
	GSE42861	450K	[17]	Peripheral blood from 354 rheumatoid arthritis patients and 335 controls.	689	[**control**|arthritis]
Breast Tum	GSE32393	27K	[35]	Breast tumor samples: 91 invasive ductal, 13 invasive lobular, 10 mucinous or medullary; 76 were ER+.	114	[**ER-**|ER+] + [**duct**|lob|muc or med] + age
Gastric	GSE30601	27K	[53]	203 gastric tumors and 94 matched gastric non-malignant samples.	297	[**normal**|tumor]
Liver	GSE60753	450K	[54]	34 normal liver tissues, 21 cirrhotic tissues (due to alcoholism), 45 cirrhotic tissues [due to chronic hepatitis B (HBV) or C (HCV) viral infection].	100	[**normal**|CirrEtOH|CirrV]
Artery	GSE46394	450K	[55]	15 normal aortic tissues, 15 atherosclerotic lesions, 19 carotid atherosclerotic samples.	49	[**normal**|ath|carotid ath] + [**female**|male] + age

* For the HNSCC data, age is not available in the GEO submission GSE30229, but was obtained from the authors of the original study.

**Figure 1:**
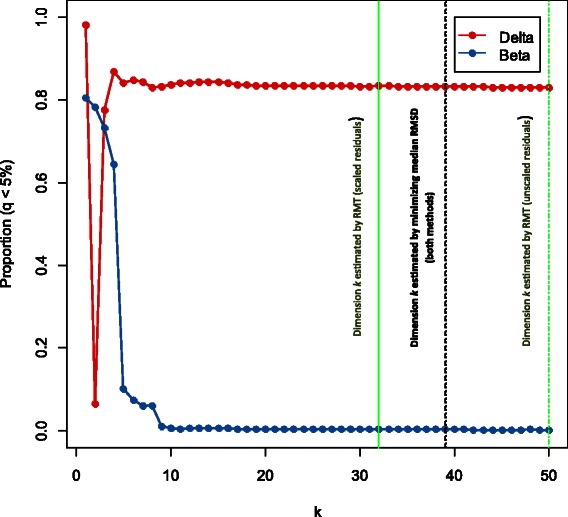
**Significance profiles for rheumatoid arthritis case vs. control coefficients.** Varying the value of the dimension parameter *k* results in differing numbers of significant Δ_*k*_ and **B**_*k*_ coefficients, with stablilization of results for *k* ≥ 10. Similar plots for the other eight data sets are given in the Additional file 1.

We hypothesized that for sufficiently large values of *k* << *n*, the number of significant (*q* < 0.05) **Δ**_*k*_ loci will generally be larger than the number of significant **B**_*k*_ loci. Figure 1 demonstrates that for the 450K rheumatoid arthritis data set, the number of significant (*q* < 0.05) **Δ**_*k*_ and **B**_*k*_ coefficients stabilized after about *k* ≥ 10. All methods of estimating dimension returned values that were in the stable region, although the values themselves were different. In the stable region, the number of significant **B**_*k*_ coefficients was vastly smaller than the number of significant **Δ**_*k*_ coefficients, although still numbering over 1000 (as is shown in Additional file 1: Figure S1). This pattern held true generally over other data sets (see Additional file 1: Figures S2 – S9). The one exception was the artery data set, which exhibited instability for *k* ≥ 25; however, this data set had small sample size relative to the other data sets, with only 44 residual degrees of freedom in the error terms (rows of the residual matrix **R**). Indeed, the procedure broke down after *k* ~ 40. However, reasonable stability was evident around *k* = 10, so we chose this value for subsequent analyses. We remark that for most analyses the fraction of significant **Δ**_*k*_ coefficients was typically close to the number of significant **A** = **Β**_0_ coefficients (Additional file 1: Figure S10), representing the total effect of the phenotype, though this fraction was rarely identical. Although the number of significant **B**_*k*_ coefficients was generally much smaller than the number of significant **Δ**_*k*_ coefficients, it was almost never zero at selected values of *k* (see Additional file 1: Figure S1), with the one exception of age coefficients in the ovarian cancer case/control data set (age in this data set was omitted from subsequent gene-set analysis because of the absence of loci with significant *q*-values). As shown in Additional file 1: Figure S11, the two methods of selecting *k* proposed in this article resulted in about the same order-of-magnitude in estimating median root-mean-square-difference (RMSD, the objective statistic used to measure stability at a value of *k*), and often much smaller order-of-magnitude than the random matrix theory method. Thus the proposed methods typically chose “flatter” regions of *k*.

To assess the biological significance of results, we selected several gene sets and investigated the enrichment of significant (*q* < 0.05) loci within each set. We hypothesized that for *k* selected by our proposed method, significant **Δ**_*k*_ coefficients will over-represent in gene sets that are reasonable for the target tissue, and in particular, significant **Δ**_*k*_ coefficients in blood data sets will be enriched for CpGs that are known to differentiate major types of leukocytes, i.e. DMRs for blood. Additional file 1: Figure S12 shows the results of analysis of DMR sets for the seven non-reference data sets and two reference data sets. Strongly significant enrichment of DMRs within **Δ**_*k*_ coefficients was evident for all comparisons except gastric tumor vs. normal. Enrichment of DMR gene sets for reference sets is unsurprising since these reference sets were used to determine the DMRs. Enrichment of DMR gene sets makes sense for data sets where blood was analyzed, since the DMR loci are precisely those that differentiate blood cell types. It also makes sense for breast tumors, as tumor infiltration by leukocytes has been well established [28], for liver tissues, where there is an inflammatory response in alcohol-related disease and an antiviral immune response in infection-related disease [29], and for artery, where leukocyte infiltration has been observed in atherosclerosis [30,31].

Additional file 1: Figure S12 also shows analysis of polycomb group target (PcG) sets. Polycomb group target regions represent sites of binding and occupancy of polycomb group repressor complexes, which play a critical role in defining cell-type specific expression patterns [32,33] and which may represent regions targeted for epigenetic variation resulting in altered cellular function [34]. Thus we hypothesized that those loci driving cellular composition effect are over-represented by regions considered PcG targets. PcG gene sets were significantly enriched for all **Δ**_*k*_ coefficients, though weakly in the age association for head and neck squamous cell carcinoma (HNSCC). PcG gene sets were weakly enriched for significant **B**_*k*_ coefficients in the following comparisons: atherosclerotic lesion vs. normal aorta, atherosclerotic carotid tissue vs. normal aorta, and age (in HNSCC data set), as well as for some of the cell types in the reference data sets. Given that PcG targets are deeply involved with the development and maintenance of hematopoietic stem cells, as well as development of neoplastic tissues, the enrichment of PcG genes among most of the comparisons we investigated is not unreasonable. In particular, the profound over-representation of PcG targets in the significant **Δ**_*k*_ coefficients from the breast tumor data is reassuring, as the original analysis of this data demonstrated such over-representation amongst loci exhibiting differing methylation between tumors and normal tissues in a variety of women’s cancers [35].

**Figure 2:**
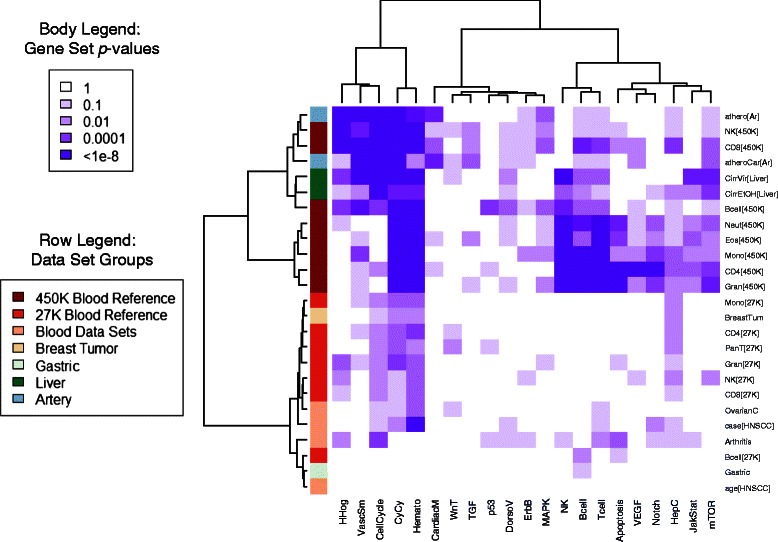
**Gene-set p-values for KEGG pathways and Δ**_*k*_ **coefficients.** The clustering heatmap shows gene set *p*-values depicted by color, with data set indicated in the row annotation track. Clustering was achieved by applying a Euclidean metric to – log_10_ *p*-values and using Ward’s linkage method. Note that the gene set tests were conducted as exact Mantel-Haenzel tests, stratified by CpG Island status (27K) and by Infinium biochemistry type, relation to CpG Island, and gene region (450K).

In Figure 2, a clustering heatmap summarizes the gene set results for **Δ**_*k*_ and Kyoto Encyclopedia of Genes and Genomes (KEGG) pathways, with clusters obtained by Euclidean metric and Ward’s method of clustering applied to log-significance. In general, 27K and 450K data sets clustered together, as one would expect from the different numbers of loci on each array platform. However, the arthritis case/control data set clustered together with the other two blood case/control data sets and the 27K reference data coefficients within the 27K cluster. The non-blood 450K data sets clustered with the B-cell, CD8, and NK coefficients from the 450K reference data set, while the remaining 450K reference data coefficients clustered together in a separate cluster. In general, the 450K data set cluster showed enrichment in the *cytokine-cytokine receptor interaction* and *hematopoietic cell lineage* pathways. The cluster containing only 450K reference coefficients showed additional enrichment for the three lymphocyte-specific-signaling pathways (*B-cell*, *NK*, and *T-cell*), which were less strongly enriched for the coefficients in the other 450K cluster. Note that the *vascular smooth muscle contraction* pathway was strongly enriched for the arterial data set and for cirrhosis associated with viral infection. Interestingly, the most striking difference between the two cirrhosis coefficients was the lack of significance of *Hepatitis-C* pathway in the coefficient representing differences between virally-associated cirrhosis and normal liver! However, this is explained by the enrichment of the *Hepatitis-C* pathway by significant **B**_*k*_ coefficients (*p* = 0.0028 for cirrhosis related to viral infection vs. *p* = 0.80 for alcohol-related cirrhosis), suggesting DNA methylation differences not mediated by cell-composition effects. Additional file 1: Figure S13 shows a similar clustering heatmap for **B**_*k*_ coefficients. Gene set enrichment was much weaker, occurring primarily for hematopoietic pathways and reference lymphocyte coefficients. Interestingly, the arthritis case-control coefficient showed significant enrichment of **B**_*k*_ coefficients in these pathways. Odds ratios for enrichment of gene sets by significant loci are illustrated by clustering heatmaps in S14 (**Δ**_*k*_) and S15 (**B**_*k*_).

Additional file 1: Table S1 provides a list of genes mapped to the CpGs having significant (*q* < 0.05) **B**_*k*_ coefficients, or the top 50 such CpGs. While it is not our purpose to present an exhaustive epigenetic analysis of each data set, we note some features of these lists to demonstrate the biological plausibility of the cell-mixture-adjusted hits. For example, the two top hits for arthritis case vs. control were mapped to the NLRC5 gene, which has recently been implicated to play a key role in antigen presentation [36] and inflammasome activation [37], suggesting a potential link to inflammation present in rheumatoid arthritis. Genes among the top hits for breast cancer ER+ vs. ER- are related to transcription (BCOR, CST11, HERC2, HEYL, IGF2BP3, MBP, NRIP2, RASSF5, SCML4, SMARCC2, SMPX, SSX1, TBX19, VHL, ZBTB16, ZNF124, ZNF560) and signal transduction (ARHGAP27, CHRNB4, KALRN, LRP12, PDCL, TNFSF18), which may be important pathways in breast tumorogenesis [38]. Many genes among the top hits for gastric tumor vs. normal gastric tissue are related to cancer and tumorogenesis (ASL, BCL2L2, CASP1, IFITM1, IL11RA, LCAT, LRIG1, NFE2L2, PABPC1, PFN1, PPAT, PSMD2, RANBP2, RARG, TERF2IP, THOC1, UHRF1). While extensive efforts would be required to confirm the phenotypic associations with these individual genes, they demonstrate the biological plausibility of significant **B**_*k*_ coefficients.

We also evaluated the behavior of the reference-free method in various null scenarios. Additional file 1: Figure S16 summarizes the results. In all three scenarios (completely null by permutation, or null-linear with non-null nonlinear effects of two different magnitudes) there were no significant (*q* < 0.05) **Δ**_*k*_ coefficients for *k* ≥ 10. For the completely null scenario, none of the **B**_*k*_ coefficients were significant. For the two nonlinear scenarios, the proportion of significant **B**_*k*_ coefficients was substantially higher than for the original analyses upon which these null scenarios were based, with the higher magnitude effects resulting in a higher proportion of significant **B**_*k*_ coefficients. In summary, the reference-free method appeared to differentiate nonlinear effects from those that act together in a linear fashion.

**Figure 3:**
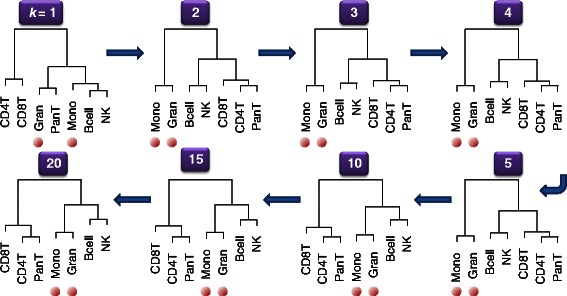
**Sequence of clustering dendrograms from 27K blood reference data set.** The dendrograms display the results of clustering the non-intercept columns of the coefficient matrices Δ_*k*_ by applying Euclidean metric and Ward’s linkage method. Note that the intercept (reference) represented whole blood. The sequence shows that differing the values of *k* results in distinct levels of information with respect to cell lineage. Red dots indicate myeloid lineage (granulocyte and monocyte).

In Methods we argue that a recursive partitioning principle holds for DNA methylation profiles, i.e. that the mean methylation profile for different cell types will partition in a manner consistent with cell lineage. To demonstrate this we applied hierarchical clustering to coefficients from the two reference data sets. Figure 3 shows the results of clustering ${\boldsymbol{\Delta}}_k^T$ coefficients from the 27K reference data set at different values of *k*. The figure demonstrates that for *k* = 1, the coefficients in ${\boldsymbol{\Delta}}_k^T$ did not respect the myeloid/lymphoid lineage distinction, but for 2 ≤ *k* ≤ 5, the split occurred exactly as one would expect. For *k* ≥ 10, Natural Killer (NK) and B cells were grouped more closely with granulocytes and monocytes than with other lymphocytes, although they were still separated from the myeloid lineage. This demonstrates the potential addition of noise when including additional terms into ${\boldsymbol{\Delta}}_k^T$. Additional file 1: Figure S17 similarly shows that for the 450K data set, the myeloid/lymphoid distinction was respected for *k* ≥ 1, but that the relative groupings of myeloid types (especially eosinophils) changed depending on the value of *k*.

The reference-free method relies on applying a SVD to the matrix obtained by concatenating the total effects matrix **A** with the corresponding residual matrix **R**. We have argued that the resulting left singular vectors **u**_*k*_ corresponding to the largest eigenvalues represent variation among constituent cell types. As an evaluation of the hypothesis that the left-singular vectors **u**_*k*_ of the SVD in (4) concentrate a majority of information about major cell types within the initial values of *k*, we computed the cross products of the left-singular vectors **u**_*k*_ across all five 27K data sets. We hypothesized that because the variation in the blood data sets are based on the same major cell types, for lower values of *k*, left-singular vector cross-products among blood will have higher absolute values than cross-products between blood and non-blood tissues and among non-blood tissues. The clustering heatmap in Additional file 1: Figure S18 depicts the absolute values of the cross-products in left-singular vectors **u**_*k*_ among the 27K data set analyses. Clustering was based on Euclidean metric and Ward’s linkage. Note that because the vectors must be orthogonal within a single data set, tight clusters will not have vectors from the same data set. As shown in the figure, the cross-product absolute values did appear to cluster together more tightly for lower values of *k* than for higher values. In particular, the first vector from each data set appeared in the lower-right cluster, except for the gastric tissue data set for which the second vector was included in the cluster; this large cluster split into two smaller clusters that were composed entirely of blood or entirely of non-blood tissue. Another large cluster contained one vector (*k* ≤ 2) from each data set and, additionally, the third vector from the HNSCC data set, with each 2-term subcluster containing only blood or only non-blood tissues. The remaining clusters were smaller and showed weaker clustering. This result is inconclusive in itself, since the first term from each data set appeared in one cluster and could represent simply known variation, e.g. differences across CpGs with different CpG Island status. However, we also clustered the intercept columns of each **Δ**_*k*_ matrix across 27K data sets and across 450K data sets, and finally computed stratified correlation coefficients among intercept columns. Again, our hypothesis was that for **Δ**_*k*_, blood data sets would cluster together relative to other tissues and exhibit stronger correlation, while for *k* > 0, no such clustering or correlation would exist for **B**_*k*_ coefficients. Additional file 1: Figure S19 shows clustering dendrograms for the intercept column of **A** and for **Δ**_*k*_ at various values of *k*. Clusters from **A** demonstrated marked separation of blood data sets from breast tumor and gastric tissue data sets. Note that the intercept term in the 27K reference data set represented mean methylation in whole blood. Interestingly, the separation between tissue types was evident in **Δ**_2_, with only two terms from the SVD, and completely recovered by the first 10 terms with **Δ**_10_. The anticipated separation was completely broken by removing the first SVD term (resulting in **B**_1_). In other words, the well-known differences in methylation between tissue types that were evident from **A** were contained almost wholly in the first term of the SVD, i.e. a rank-1 linear combination suggestive of cell-composition effects. Additional file 1: Figure S20 shows the correlation between the intercept term in the 27K reference data set correlated strongly with the intercept terms in the other blood data sets (stratified Pearson *r* > 0.99) for moderate and high values of *k*, but never correlated with the intercepts from the other two tissues more strongly than *r* > 0.9. In contrast, the intercept terms of **B**_1_ did not correlate: stratified correlations with the intercept from the reference data set were 0.29 (HNSCC blood), 0.44 (ovarian blood), -0.06 (breast tumor), -0.06 (gastric tissue). Again, this result suggests that the initial terms of the SVD drive correlations within and across tissue types, suggestive of strong cell-composition effects. Additional file 1: Figure S21 shows clustering dendrograms for 450K, similar to what Additional file 1: Figure S19 shows for 27K. The pattern was the same except that it was necessary to remove two terms of the SVD (resulting in **B**_2_) to break the anticipated grouping between the reference blood data set and the arthritis data set. As in Additional file 1: Figure S20, Additional file 1: Figure S22-A shows correlations with 450K reference blood across different values of *k*. The pattern was more striking than in Additional file 1: Figure S20, demonstrating strong correlation between blood data sets and weaker correlation with liver and artery, a pattern that was constant across all values of *k*. Additional file 1: Figure S22-B shows a complementary pattern of weak correlation in **B**_*k*_ intercepts across all data sets.

### Discussion of empirical results

The novel linear expansion of DNA methylation array presented here illustrates that tissue differences and tissue-specific cell lineages are evident in only a few linear terms of the SVD. It also suggests that principal variation driven by linear combinations contains a majority of the biological information in DNA methylation data sets. This finding is in line with our prior work demonstrating that phenotype associated methylation profiles are driven by variation in the underlying composition of major types of cells within the sample [14-16]. Our present work also suggests that this finding may apply more widely in studies that utilize target tissue samples other than peripheral blood. It is also consistent with other recently published work that demonstrates the ability to deconvolve DNA methylation data to obtain underlying information about cell mixtures [22,39].

Our analysis separates composition effects by major cell types from subtler effects within a cell type or focused effects across cell type. This was clearly apparent when examining gene set enrichment of the significant **Δ**_*k*_ coefficients arising from the blood cell reference datasets, which showed the predicted highly significant over-representation within demonstrated blood cell type DMRs (which had in fact been chosen on the basis of these data sets). This over-representation was also seen in data from other studies using peripheral blood, including the ovarian cancer, rheumatoid arthritis, and HNSCC case-control analyses. Interestingly, other than the gastric tissue data set, the other data sets demonstrated significant over-representation of DMR loci among significant **Δ**_*k*_ coefficients. This is potentially due to immune cell infiltration or inflammatory process, as we suggest above. In most of the datasets the set of significant CpGs showed enrichment for PcG targets, important for cell growth and differentiation, again potentially reasonable for the data sets we analyzed. Several KEGG gene set analyses also showed over-representation among significant **Δ**_*k*_ coefficients of pathways such as cytokine-cytokine interactions, hematologic processes, and NK cell signaling for non-blood data sets; again suggestive of immune-cell infiltration or inflammation.

We note that the sets of significant **Δ**_*k*_ coefficients corresponded in large part to the sets of significant **A** (unadjusted) coefficients. Although it may be possible that for moderate values of *k* the reference-free method merely assigns a substantial portion of any **A** effect to **Δ**_*k*_ rather than to **B**_*k*_, the null scenarios we considered suggest instead that the method is able to correctly partition effects into linear and nonlinear categories, as was suggested by the simulations conducted in our original paper [19]. Therefore, the alternative explanation, that much of the phenotypic effect on DNA methylation is explained by latent linear variables suggestive of cell composition effects, is more plausible.

Although the principal variation in DNA methylation profiling appears to be explained through these compositional effects, there are loci that still show significant covariation with phenotype outside these predominant linear structures. We note that the reference-free algorithm has been recently applied for the specific purpose of discovering such loci, e.g. Wilms’ tumor biomarkers have been confirmed recently for significant cell-mixture adjusted coefficients obtained using our reference-free algorithm [20]. According to theoretical considerations given earlier, this variation may be interpreted as evidence for effects involving consistent but focused changes across cell type or subtly distinct forms of major cell types arising or being selected for through pathological processes. Biologically, this is an important demonstration, as it would suggest that, beyond profound alterations in major cell type composition, subtle changes to the underlying distribution of cells within tissues can be an important part of the pathological process underlying a variety of conditions. This is well understood in oncology, and even in diseases such as hepatic cirrhosis where dysplastic changes are apparent. In some cases, these changes may not be histologically obvious, but can still have important implications in disease progression. For example, the expansion of stem cell compartments in tissues such as adipose or regenerative processes in bone marrow may arise in expanded numbers of cells with bivalent chromatin, marked perhaps by 5-hydroxymethyl cytosine but without obvious histologic signs of tissue differences. Through these analytical methods, these potentially crucial and possibly prognostic or etiologically relevant alterations may be observable.

An issue that was somewhat underdeveloped in our original paper was the selection of the dimension parameter *k*. In particular, there has been some concern in response to our original paper that the choice of *k* may influence results. We had suggested the use of an established algorithm designed for an entirely different purpose, and found in this paper that it did not optimize the criterion we have found most useful for this type of analysis (see Additional file 1: Figure S11B). Although our proposed alternative by definition optimizes this criterion, both methods often resulted in values of *k* for which the solution changed very little in moving to adjacent values. This demonstrates some robustness of the reference-free method to reasonable choices of *k*, a finding that supports the use of the reference-free algorithm. However, as shown in Figure 1 and Additional file 1: Figures S2 through S8, choosing a value of *k* that is too low or too high (i.e. too close to the available residual degrees of freedom) will result in unstable partitioning of effect. Robustness to the choice of *k* also suggests that most data sets will show variation in a small number of cell types, beyond which all detectable variation is of a more subtle variety. Some biologists may be interested in using plots similar to those shown in Figure 1 and Additional file 1: Figures S2-S9 to determine the (often unknown) number of major cell types *k*′ in a tissue by finding the minimal value of *k* for which the solution seems stable. However, we note that the dimension *k* evident in a real data set may not correspond to the number of cell types in a linear fashion; this was subtly evident in Figures 3, Additional file 1: Figure S16, and S18-S21, which demonstrated that a small number of terms recovered the majority of variation in the data sets. Indeed, in some cases *k* may scale with the base-2 logarithm of the number of cell types *k*′, as each dimension is able to differentiate one lineage “split”.

We examined the over-representation of blood cell DMRs and PcG targets, as these are well-established gene sets, and in the case of blood-based analyses they are critical. For other tissues, cell-specific DMRs are less well established, but our findings linking pathology to these compositional effects points to the existence of such DMRs, and suggests an important area for further epigenetic research.

Importantly, this work also has implications for conducting an EWAS: adjusting for cell composition and/or looking for cell-composition effects (where most of the information is in the initial terms of the SVD), is crucially important for interpreting the data. For example, the distribution of cell types in numerous organs (e.g. autocrine or exocrine organs) may be affected by the conditions of organismal development. The Barker hypothesis holds that developmental effects of environmental exposure alter susceptibility to later chronic disease [40]. In this construct, maternal exposures (including diet, exercise etc.) can affect the development of fetal offspring in a manner that alters tissue composition and function. That is, alteration of the mixed cellularity of important cell types in tissues, occurring in-utero and persisting into adulthood, could be a determinant of later disease and this could be examined using these methods. Further, cell adjustment would also allow for assessment of the usually much smaller effects outside the initial terms of the SVD.

It must also be noted that adjusting for cell composition significantly complicates the process of EWAS replication and validation. Replication, of course, can be accomplished if there exists a replication population with array data; in this case the models can be re-applied. However, often the process of replication and validation is accomplished using an orthogonal approach such as pyrosequencing of selected targets, as has been recommended by some as a critical approach in EWAS quality control [41]. In this case, separation of the cellular composition effects and environmental effects would be extremely challenging without additional array-scale data.

We acknowledge one limitation in this work, the assumption that linear associations necessarily represent cell mixture effects. While it is true that cell mixture effects *must* be linear, the converse may not be true, i.e. there may be processes outside cell-composition effects that result in linear associations. We have not ruled out the well-known potential for approximately linear technical artifacts; although we did not correct for them in this analysis due to incomplete access to the necessary low-level files, we emphatically recommend the use of accepted pre-processing techniques such as *BMIQ* or *FunNorm* for 450K [42] before applying reference-free methods in the analysis of single data sets. Even without adjustment for technical effects, we saw evidence in our empirical analyses of truly biological information present in the initial linear terms of the SVD. Given that most biological processes are nonlinear, it would be difficult to imagine a theoretical basis for linearity of truly epigenetic effects arising *outside* of cell-composition. Of course it is possible that a strong nonlinear effect would result in strong first-order linear terms that might be interpreted as cell-composition effects, but such widespread systematic effects would likely represent a selection process that is ultimately cell-mediated, i.e. occurring outside the cell nucleus. An example of this is the gastric tissue data set, where the profound difference in behavior between normal gastric tissue and tumor ultimately results from the selection of tumor cells over normal cells. We do not rule out the potential for widespread intra-nuclear processes occurring sporadically (as opposed to systematically) but would argue that such effects, if not resulting in the expansion of an altered type via selection process, would present as a more random process, such as those targeted by algorithms such as EVORA [43,44]. We note that similar methods such as SVA and ISVA [23,24] may be used for the purpose of cell-mixture adjustment, but because they do not use the cell-mixture information available in the regression coefficients, they may perform less adequately for this purpose, as we have shown previously [19]. Although we argue in this paper that the most likely interpretation of linear associations is that of cell composition, a more general point can be made: that the various interpretations assigned to phenotypic associations with DNA methylation require greater scrutiny than has often been offered in many EWAS reports.

## Conclusions

In this article we have presented a mathematical basis for reference-free cell-mixture adjustment, arguing that the total effect of a phenotype on DNA methylation can be decomposed into orthogonal components, one representing the effect of phenotype on proportions of major cell types, the other representing subtle differences and global effects at focused loci. Using nine different DNA methylation data sets arrayed on Illumina Infinium platforms, we have demonstrated empirically that a majority of the information regarding lineage and cell-type appears to reside in the first few terms of an orthogonal linear expansion, thus corroborating the assertion that these initial principal components should be interpreted as cell composition effects. We also demonstrate that the remaining terms nevertheless may contain significant effects at a small number of loci, and these effects may represent either subtle alterations in cell type or focused changes common to all cell types. In addition, we demonstrate reasonable robustness to the choice of *k*, the number of terms to interpret as effects of major cell types, and present a method that is designed to find the value of *k* that results in minimal change in results across adjacent values.

Taken together, our work demonstrates that reference-free algorithms for cell-mixture adjustment can produce biologically valid results, separating cell-mediated epigenetic effects from those that are not mediated by major cell types, and thus represents a useful method for distinguishing the two types of effects in EWAS. In general, we argue that the biological interpretation of epigenetic associations evident from DNA methylation data requires closer examination than has often been offered in EWAS reports.

## Methods

### Novel methods for estimating dimension

We have previously proposed using a random matrix theory approach for estimating *k*, demonstrating that it performs well under simulation in small artificial data sets [19]. The method was originally proposed for determining the number of singular values to keep in *isva*, an approach that is similar but does not use **A** as part of the matrix to be decomposed [24]. In essence, the method looks for the smallest number of singular values of **R** (standardized by row) for which the residual matrix (analogous to **B**_*k*_ above) is consistent with a randomly generated matrix. On the other hand, an empirically useful criterion for choosing *k*, based in part on biological considerations, is to find values of *k*, such that terms *λ*_*j*_ **Q**_*j*_ are small relative to **Δ**_*k*_ for *j* close to *k*. The biological interpretation is that such values of *k* effectively exhaust the variation among major types of cells, and represent a “stable” solution. We propose to operationalize this as follows: for each candidate value of *k*, compute the *median*-*root-mean-squared-difference* between successive values of **Δ**_*k*_, $RMS{D}_k= me{d}_h{\left\{{\left(d-1\right)}^{=1}{\lambda}_j{\displaystyle {\sum}_{l=2}^d{Q}_{hl}^2}\right\}}^{1/2}$, where each sum-of-squares is computed separately for each row over the non-intercept column entries *l* of **Q**_*j*_ and the median is computed over the rows *h*; *k* is then chosen as the value that minimizes this median statistic.

We previously proposed a bootstrap method for generating the sampling distribution of **A** and **B**_*k*_ (and therefore **Δ**_*k*_) [19]. Thus it is possible to construct t-statistics for each entry of **Δ**_*k*_, and an alternate but similar procedure could minimize the median of the row-sums of the squares of the element-wise differences of these statistics. In principle it would be better to use the formal F-statistic across all non-intercept columns, but this vastly increases the computational burden of the procedure (since it involves a large number of matrix-inversions).

### Methods for empirical evaluation

To evaluate and demonstrate the concepts proposed above, we applied the proposed method to nine DNA methylation data sets obtained from GEO, each described in Table 1. Table 1 also indicates he regression model used in each analysis. For all but two female-only data sets, sex chromosome loci were omitted. For each of the 450K data sets, 166,314 CpGs were removed due to known problems with cross-reactivity or polymorphisms [45] and loci with greater than 5 missing values were also omitted. For 27K data sets we considered a range of *k* from 1 to 25; for three of the 450K data sets we considered a range of *k* from 1 to 50, but due to small residual degrees of freedom, we considered only a range up to 30 for the Reinius 450K blood reference data set.

For each data set and for each value of *k*, we computed **B**_*k*_, **Δ**_*k*_, and their element-wise bootstrap standard errors (100 bootstrap samples for each analysis) using our previously published reference-free algorithm [19]. From estimates and standard errors we computed *p*-values, and across each column of **B**_*k*_ and of **Δ**_*k*_ we computed the corresponding *q*-values using the Bioconductor *qvalue* package (version 1.34.0). Additionally, we applied the two new proposed methods for estimating *k*, as well as two variants of the previously proposed random matrix theory approach, one that scales the rows of **R**, and one that does not.

To assess the biological significance of results, we selected several gene sets and investigated the enrichment of significant (*q* < 0.05) loci within each set. For each data set, we used the value of *k* selected by our proposed method, except for the artery data set where we used *k* = 10 due difficulty in estimating *k*, caused by small sample size. The first gene set consisted of known DMRs for leukocytes: for 27K, the 500 CpG sites published for 27K in our earlier paper [18], and for 450K, 417 CpGs not excluded from the top 600 CpGs reported by Jaffe & Irrizary [46] based on the Reinius data set [47]. The second was a set of CpGs mapped to polycomb target genes, compiled from four separate published articles [48-51] and used extensively in our previous work. The remaining gene sets were selected KEGG pathways, obtained via Bioconductor annotation packages for the 27K and 450K platforms. Please see Table 2 for a summary of pathways investigated. To test over-representation while circumventing known problems with the application of such gene-set analysis to DNA methylation data, we used the exact Mantel-Haenzel test to stratify by genomic context. For 27K we stratified by CpG Island status, and for 450K we stratified by strata defined by Infinium chemistry type, relation to CpG Island, and gene region. Since a single CpG may be mapped to different region designations due to splice variants or gene adjacency, we used the following rule to establish precedence: promoter (“TSS1500” or “TSS200”) were combined as “TSS” and had highest precedence, and the remaining chain of precedence was as follows; TSS > 1stExon > Body > 5’UTR > 3’UTR > null. In cases where sparsity prevented stratification (a few analyses with PcG gene set) we used the unstratified Fisher test.

**Table 2:** **Gene sets analyzed**

**Abbrev**	**Description or KEGG pathway**		**Total # of CpGs**		
			**27K (all)**	**27K (auto)**	**450K (pass)**
	Total CpGs included in analyses		27578	26,486	319,264
DMR	500 previously published leukocyte DMRs [27K] [18] or overlap with 600 previously published leukocyte DMRs [450K] [46]		500	500	417
PcG	Polycomb targets compiled from four sources [48-51]		3614	3523	41,942
Apoptosis	04210	Apoptosis	157	149	1126
Bcell	04662	B cell receptor signaling pathway	138	136	1168
CardiacM	04260	Cardiac muscle contraction	121	116	967
CellCycle	04110	Cell cycle	250	245	2028
CyCy	04060	Cytokine-cytokine receptor interaction	437	426	2088
DorsoV	04320	Dorso-ventral axis formation	38	38	565
ErbB	04012	ErbB signaling pathway	154	148	1470
Hemato	04640	Hematopoietic cell lineage	153	152	779
HepC	05160	Hepatitis C	209	205	1536
Hhog	04340	Hedgehog signaling pathway	92	90	1090
JakStat	04630	Jak-STAT signaling pathway	254	250	1579
MAPK	04010	MAPK signaling pathway	469	457	4535
mTOR	04150	mTOR signaling pathway	92	88	1117
NK	04650	Natural killer cell mediated cytotoxicity	219	215	1530
Notch	04330	Notch signaling pathway	67	67	1097
p53	04115	p53 signaling pathway	144	142	1034
Tcell	04660	T cell receptor signaling pathway	191	187	1568
TGF	04350	TGF-beta signaling pathway	154	154	1314
VascSm	04270	Vascular smooth muscle contraction	193	189	1903
VEGF	04370	VEGF signaling pathway	131	131	1104

We also evaluated the behavior of the reference-free method in null scenarios. Although our original paper conducted simulations using artificial data sets with only 1000 CpGs, we devised simple but more realistic approach based on real 27K data sets. From the HNSCC and ovarian cancer case/control data sets, we obtained three null data sets as follows. First, to simulate a completely null result, we permuted the phenotype information (case status and age) with respect to the array data. Second, to simulate a null linear result with some non-null, nonlinear effects, we selected CpGs with *q* < 0.0001 for the case coefficient in a *limma* analysis, unadjusted for cell-type, conducted on an *M*-value (logit-beta) scale (458 CpGs for the HNSCC data set and 817 for the ovarian cancer data set), we then multiplied the corresponding coefficients on *M*-values by random scalars drawn from a normal distribution with standard deviation 2, and finally applied the corresponding effects to *M*-values whose case effect had been removed (on a beta scale so that any linear age effect would be maintained), converting the result back to beta scale. Note that this approach would produce a data set with weak linear age effects, no linear case effects, and nonlinear case effects at a small fraction of CpGs. The third approach was identical to the second except that a threshold of *q* < 0.001 was used (resulting in 643 CpGs for the HNSCC data set and 1163 for the ovarian cancer data set). Each of these three approaches was applied five separate times, with subsequent analysis by the reference-free method across values of *k* ranging from 1 to 25, and tabulation of the resulting number of significant (*q* < 0.05) coefficients. Our hypothesis was that the first set of analyses would produce very few significant *q*-values for either **Δ**_*k*_ or **B**_*k*_, and that the latter two sets would produce some significant *q*-values for the **B**_*k*_ coefficients but few to none for the **Δ**_*k*_ coefficients.

In order to illustrate the principle of recursive partitioning above, we conducted an additional analysis on the two reference data sets. For each value of *k*, we applied hierarchical clustering to ${\boldsymbol{\Delta}}_k^T$ (i.e. clustered the columns of **Δ**_*k*_), using Euclidean metric and Ward’s method of clustering. We have demonstrated that Ward’s method performs similarly to the recursively partitioned mixture model [27]. We hypothesized that even with relatively small values of *k*, phylogenetic relationships between cell types will be evident in the resulting clusterings.

Finally, as an evaluation of the hypothesis that the left-singular vectors **u**_*k*_ of the SVD in (4) concentrate a majority of information about major cell types within the initial values of *k*, we computed the cross products of the left-singular vectors **u**_*k*_ across all five 27K data sets and across all four 450K data sets. We also clustered the intercept columns of each **Δ**_*k*_ across 27K data sets and across 450K data sets, and computed correlation coefficients stratified over CpG Island status (27K) or more general genomic context (450K) as in the aforementioned Mantel-Haenzel test. We performed a similar analysis for the **B**_*k*_ coefficients.

The core engine of the methods discussed in this article and published in our previous work are available in the CRAN/R package *RefFreeEWAS*. The novel elements proposed in this article are available in the Additional file 1.

## Availability of supporting data

As described in Table 1, data used in this article were obtained from Gene Expression Omnibus, accession numbers GSE39981, GSE35069, GSE30229, GSE19711, GSE42861, GSE32393, GSE30601, GSE60753, and GSE46394.

## Additional file

Additional file 1: **Supplementary Materials.**

## Abbreviations

450K: Human methylation450 platform
CpG: Cytosine-phosphate-guanine dinucleotide
DMR: Differentially methylated region
EWAS: Epigenome-wide association study
GEO: Gene expression omnibus
HBV: Chronic hepatitis B viral infection
HCV: Chronic hepatitis C viral infection
HNSCC: Head and neck squamous cell carcinoma
ISVA: Independent surrogate variable analysis
KEGG: Kyoto encyclopedia of genes and genomes
NK: Natural killer cell
PcG: Polycomb group target
SVA: Surrogate variable analysis
