# Supplementary material for: Cell-composition effects in the analysis of DNA methylation array data: a mathematical perspective
Source: BMC Bioinformatics. 2015 Mar 21;16:95. doi: 10.1186/s12859-015-0527-y (PMC4392865; doi:10.1186/s12859-015-0527-y)
Supplement: Supplementary file 1 — Supplementary Materials. [file 12859_2015_527_MOESM1_ESM.docx]

**Cell-Composition Effects in the Analysis of DNA Methylation Array Data: a Mathematical Perspective**

***Supplementary Material***

E. Andres Houseman, Kelsey T. Kelsey, John K. Wiencke, Carmen J. Marsit

**Figure S1**

Number of significant $\mathbf{B}_{k}$ coefficients (q < 0.05) for various coefficients.

**Figure S2 – Significance profile for 27K blood reference data set**

|  |  |
| --- | --- |

Number of significant $\mathbf{B}_{k}$ and $\mathbf{\Delta}_{k}$ coefficients (q < 0.05) as a function of dimension parameter *k*.

**Figure S3– Significance profile for 450K blood reference data set**

|  |  |
| --- | --- |

Number of significant $\mathbf{B}_{k}$ and $\mathbf{\Delta}_{k}$ coefficients (q < 0.05) as a function of dimension parameter *k*.

**Figure S4 – Significance profile for HNSCC case/control blood data set (27K)**

|  |  |
| --- | --- |

Number of significant $\mathbf{B}_{k}$ and $\mathbf{\Delta}_{k}$ coefficients (q < 0.05) as a function of dimension parameter *k*.

**Figure S5 – Significance profile for ovarian cancer case/control blood data set (27K)**

|  |  |
| --- | --- |

Number of significant $\mathbf{B}_{k}$ and $\mathbf{\Delta}_{k}$ coefficients (q < 0.05) as a function of dimension parameter *k*.

**Figure S6 – Significance profile for breast tumor data set (27K)**

|  |  |
| --- | --- |

Number of significant $\mathbf{B}_{k}$ and $\mathbf{\Delta}_{k}$ coefficients (q < 0.05) as a function of dimension parameter *k*.

**Figure S7 – Significance profile for gastric tissue data set (27K)**

|  |  |
| --- | --- |

Number of significant $\mathbf{B}_{k}$ and $\mathbf{\Delta}_{k}$ coefficients (q < 0.05) as a function of dimension parameter *k*.

**Figure S8 – Significance profile for liver tissue data set (27K)**

|  |  |
| --- | --- |

Number of significant $\mathbf{B}_{k}$ and $\mathbf{\Delta}_{k}$ coefficients (q < 0.05) as a function of dimension parameter *k*.

**Figure S9 – Significance profile for artery tissue data set (27K)**

|  |  |
| --- | --- |

Number of significant $\mathbf{B}_{k}$ and $\mathbf{\Delta}_{k}$ coefficients (q < 0.05) as a function of dimension parameter *k*.

**Figure S10 – Proportions of significant A coefficients vs. number of significant Δ delta coefficients for non-reference data sets**

Note: A similar pattern exists for reference data sets but is not shown due to the large number of coefficients diminishing legibility.

**Figure S11 – Comparison of significance profile “flatness” in Δ coefficient obtained at dimension k chosen by various methods**

| A. Minimizing median-RMSD at estimate vs. by t-statistic | B. Minimizing median-RMSD at estimate vs. selection of *k* by random matrix theory method |
| --- | --- |

Objective function values at two proposed methods of selecting $k$; RMSD = root-mean-square-difference, as defined in the main text of article.

**Figure S12 - Significance of gene-set analyses for DMRs and PcGs**

| A. Rheumatoid arthritis vs. control (blood, 27K) | | | B. Ovarian cancer case vs. control (blood, 27K) | |
| --- | --- | --- | --- | --- |
| C. Gastric tumor vs. normal gastric tissue (450K) | | | D. Diseased liver vs. normal liver (450K) | |
| C. HNSCC case/control blood | D. Breast tumors | | | E. Arterial tissue |
| F. Reference Blood (27K) | | G. Reference Blood (450K) | | |

The bar plots show significance of gene-set results given as – log_10_ *p*-value. Note that the gene set tests were conducted as exact Mantel-Haenzel tests, stratified by CpG Island status (27K) and by Infinium biochemistry type, relation to CpG Island, and gene region (450K).

**Figure S13 – KEGG pathway p-values for B coefficients**

**
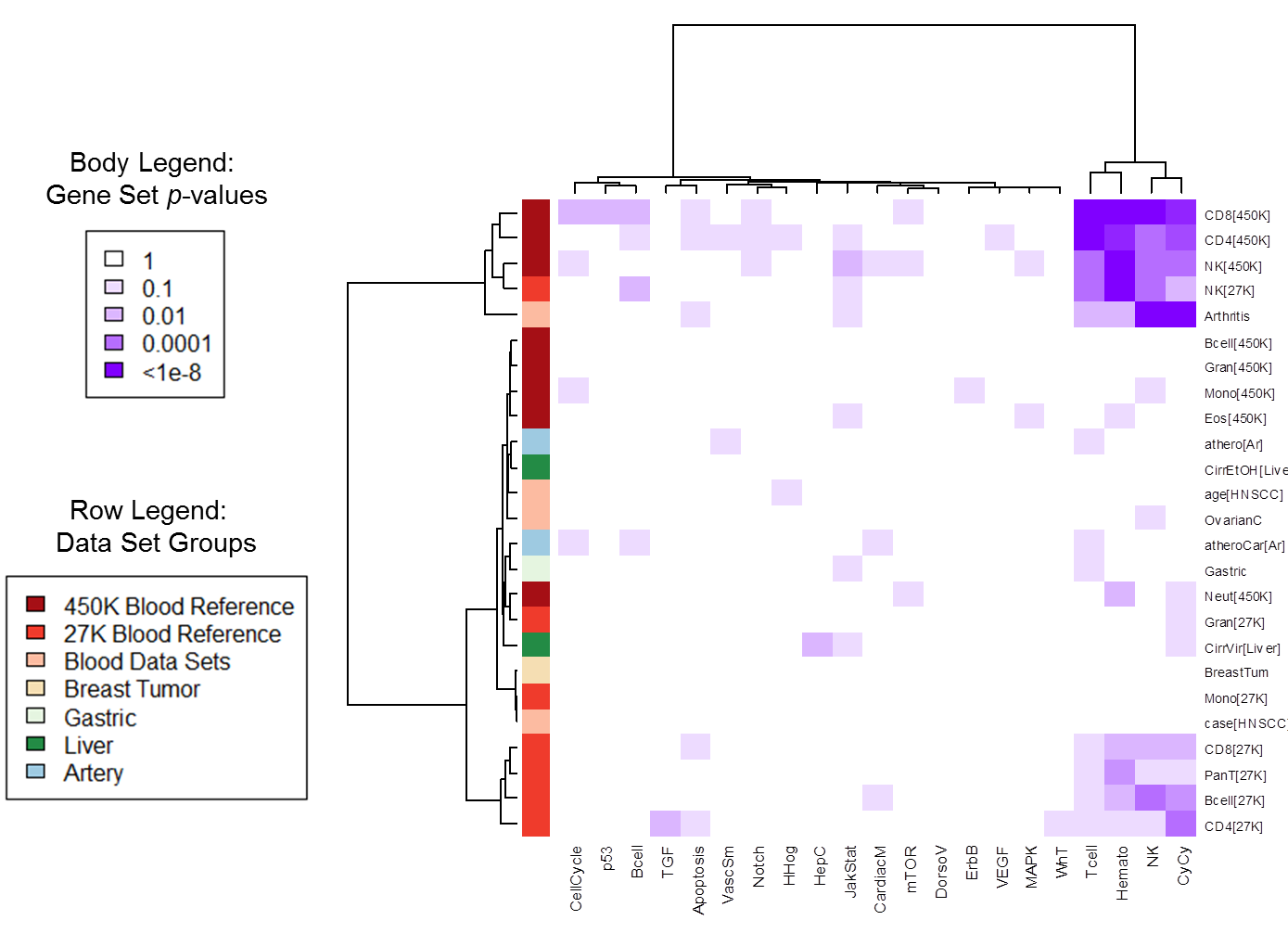
**

The clustering heatmap shows gene set *p*-values depicted by color, with data set indicated in the row annotation track. Clustering was achieved by applying a Euclidean metric to – log_10_ *p*-values and using Ward’s linkage method. Note that the gene set tests were conducted as exact Mantel-Haenzel tests, stratified by CpG Island status (27K) and by Infinium biochemistry type, relation to CpG Island, and gene region (450K).

**Figure S14 – KEGG pathway odds ratios for Δ coefficients**

**
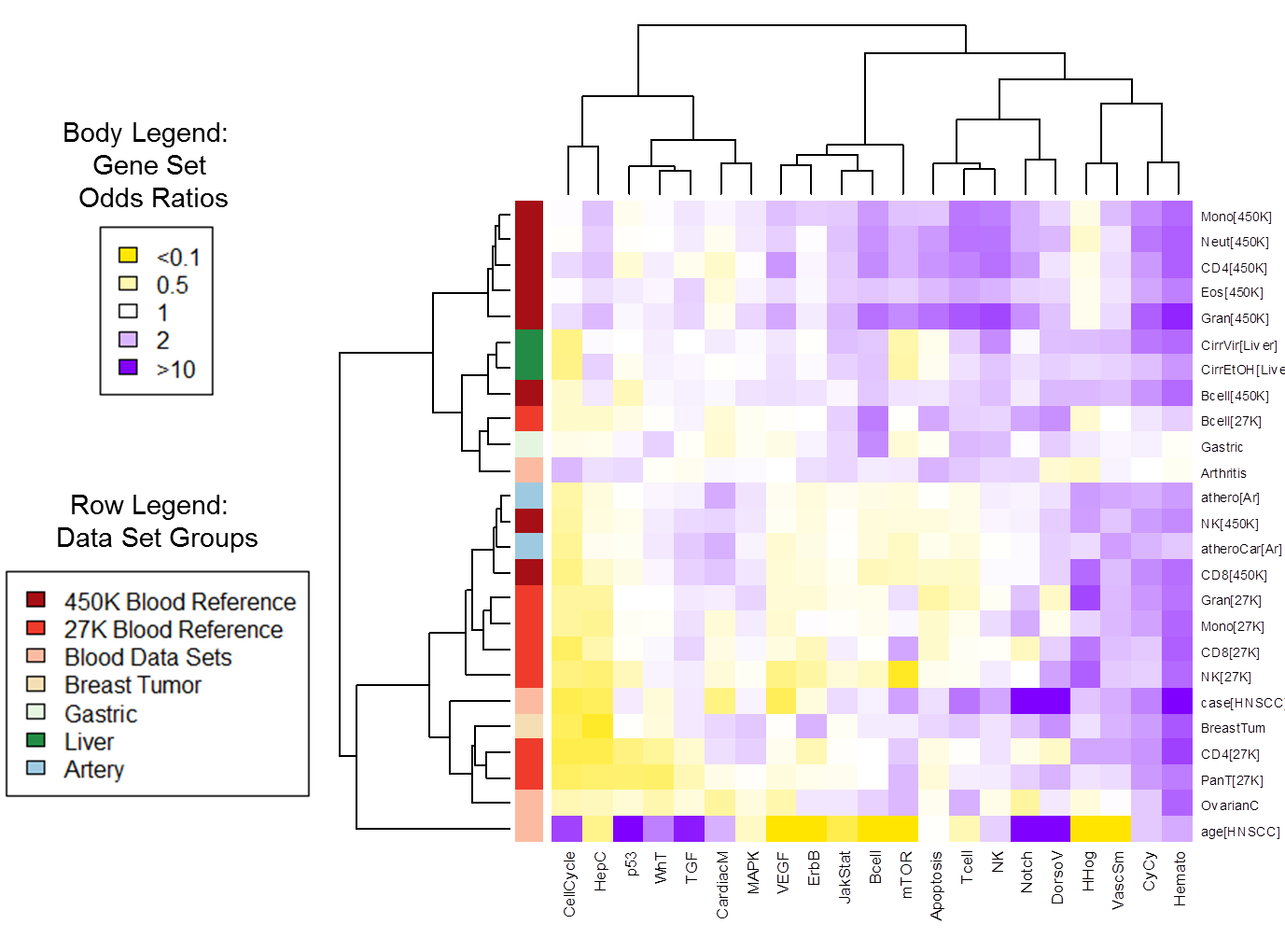
**

The clustering heatmap shows gene set odds ratios depicted by color, with data set indicated in the row annotation track. Clustering was achieved by applying a Euclidean metric to – log OR and using Ward’s linkage method. Note that the gene set odds ratios are stratified by CpG Island status (27K) and by Infinium biochemistry type, relation to CpG Island, and gene region (450K).

**Figure S15 – KEGG pathway odds ratios for B coefficients**

**
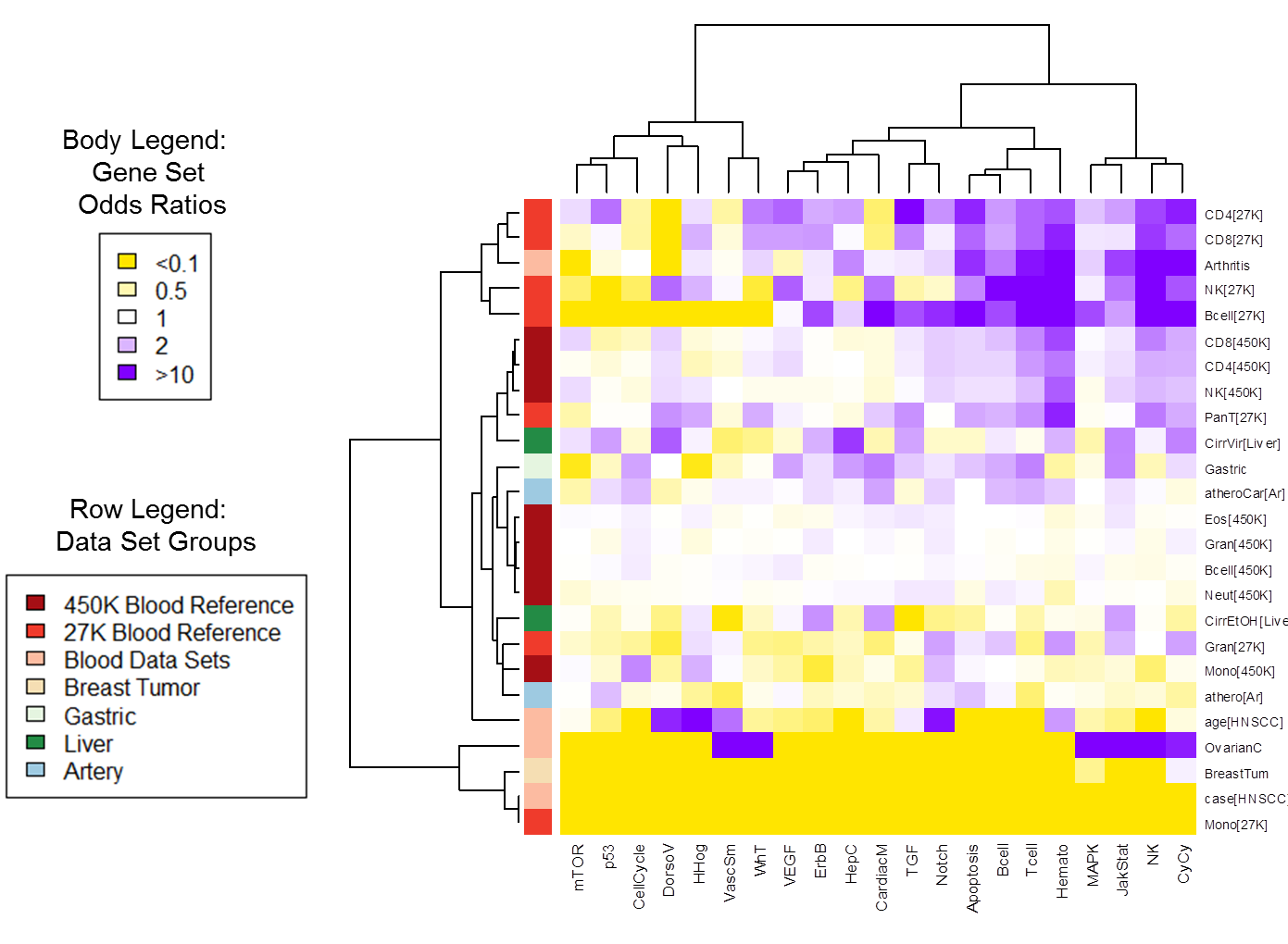
**

The clustering heatmap shows gene set odds ratios depicted by color, with data set indicated in the row annotation track. Clustering was achieved by applying a Euclidean metric to – log OR and using Ward’s linkage method. Note that the gene set odds ratios are stratified by CpG Island status (27K) and by Infinium biochemistry type, relation to CpG Island, and gene region (450K).

**Figure S16 – Analysis of data sets constructed to have null effects**

| A. Blood HNSCC (**B** coefficients only)    Note: none of the Δ coefficients were significant (*q* < 0.05) for the ranges shown in any of the three types of analyses. | B. Blood Ovarian Cancer (**B** coefficients only)    Note: none of the Δ coefficients were significant (*q* < 0.05) for the ranges shown in any of the three types of analyses. |
| --- | --- |

**Figure S17 – Sequence of clustering dendrograms from 450K blood reference data set**

**
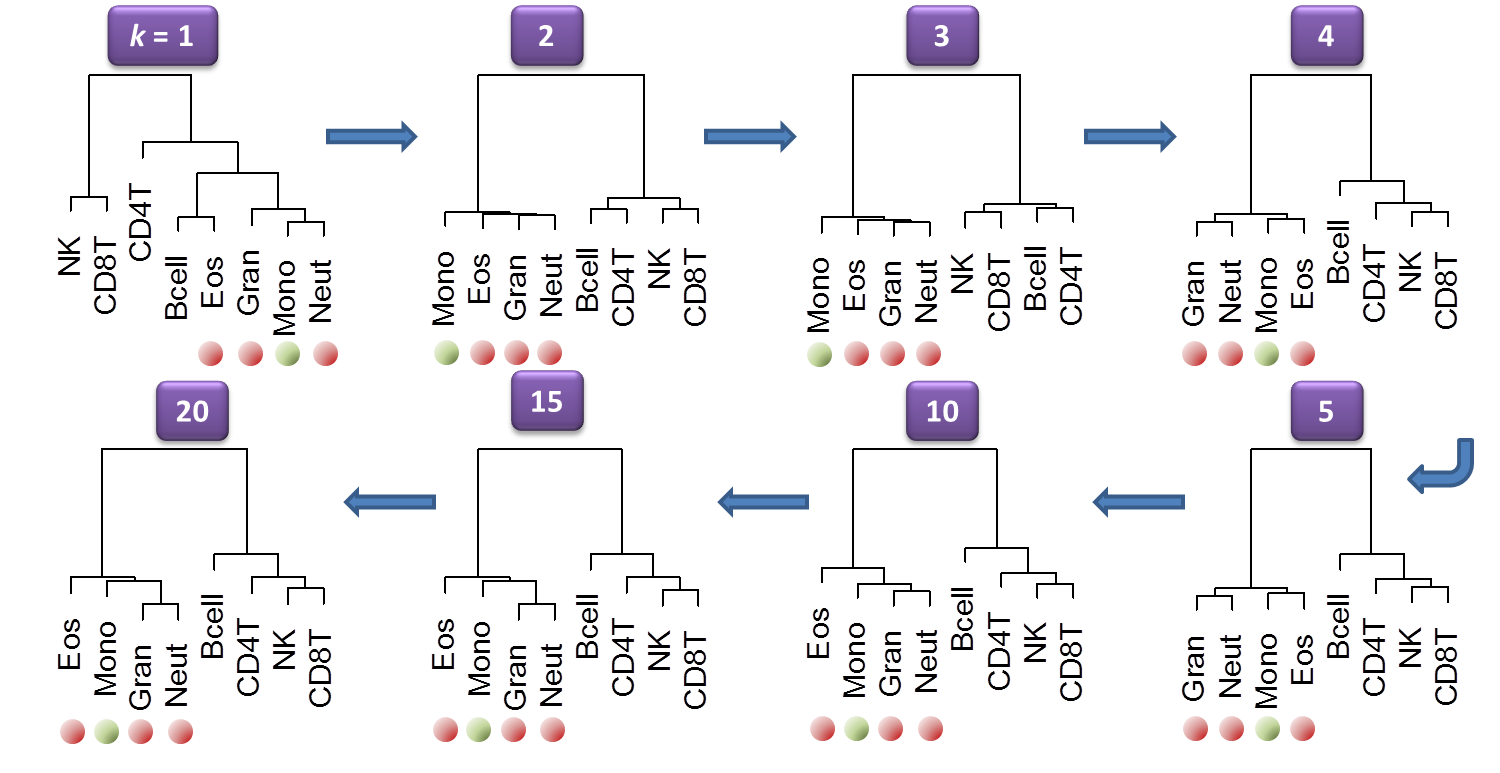
**

The dendrograms display the results of clustering the non-intercept columns of the coefficient matrices $\mathbf{\Delta}_{k}$ by applying Euclidean metric and Ward’s linkage method. Note that the intercept (reference) represented whole blood. The sequence shows that differing the values of $k$ results in distinct levels of information with respect to cell lineage. Red dots indicate myeloid lineage (granulocyte and monocyte).

**Figure S18 – Cross products of left-singular vectors from singular value decomposition**


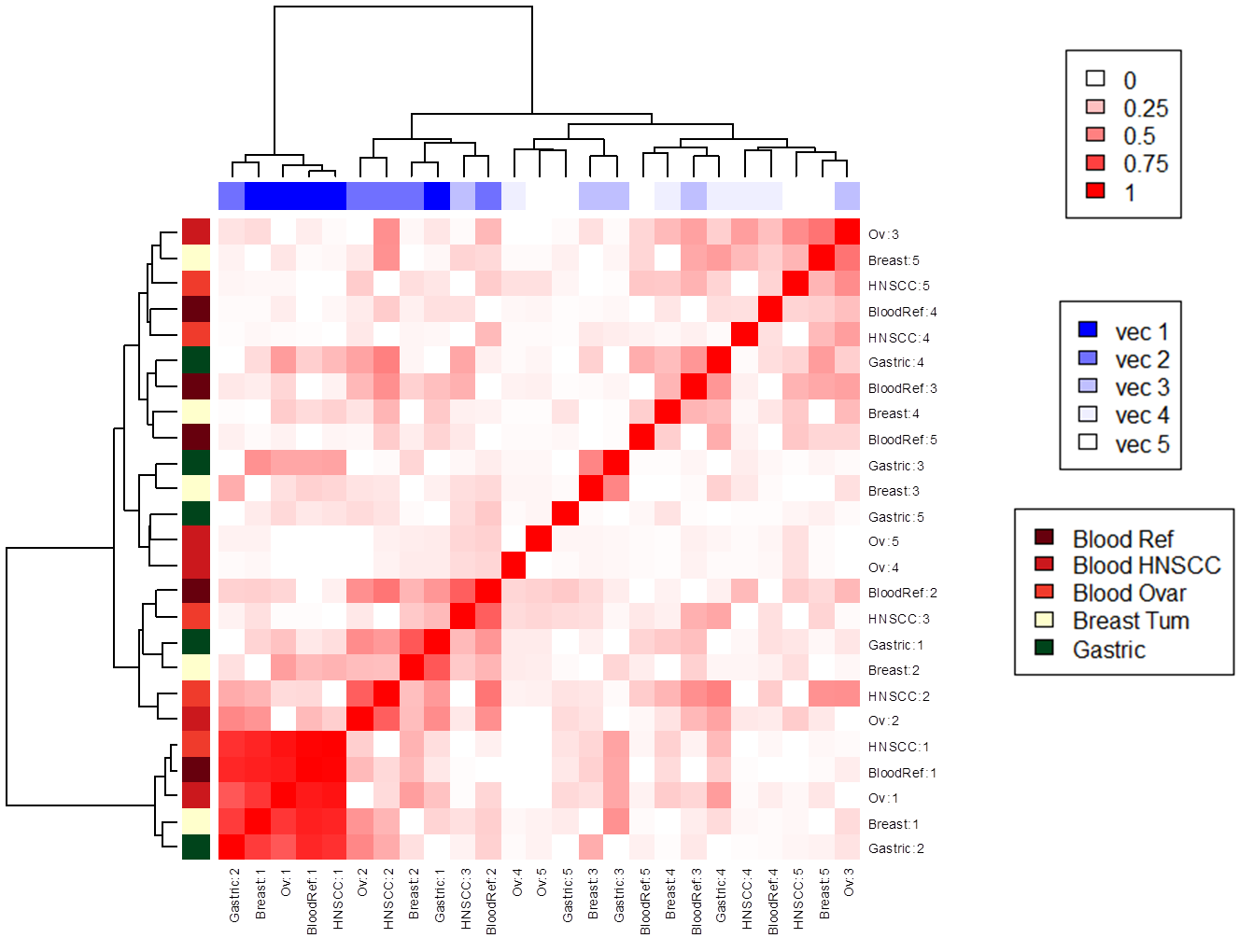


Absolute values of the cross-products in left-singular vectors $\mathbf{u}_{k}$ among the 27K data set analyses. Clustering was based on Euclidean metric and Ward’s linkage.

**Figure S19 – Clustering of Δ and B intercept coefficients from 27K analyses**

| A. Total effect (**A**)  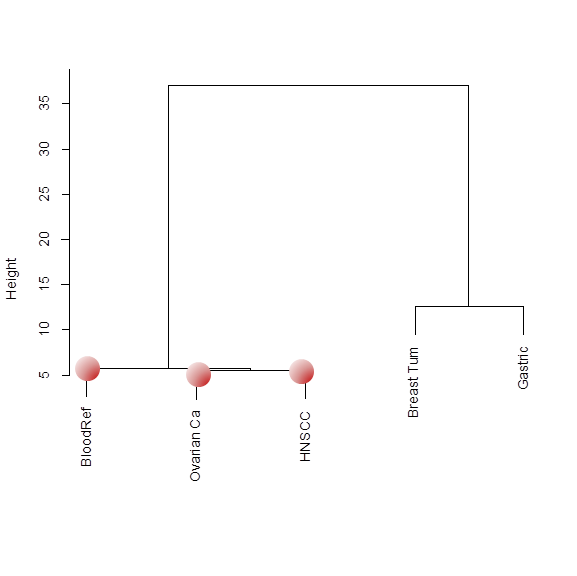 | B. First two terms of expansion (**Δ**_2_)  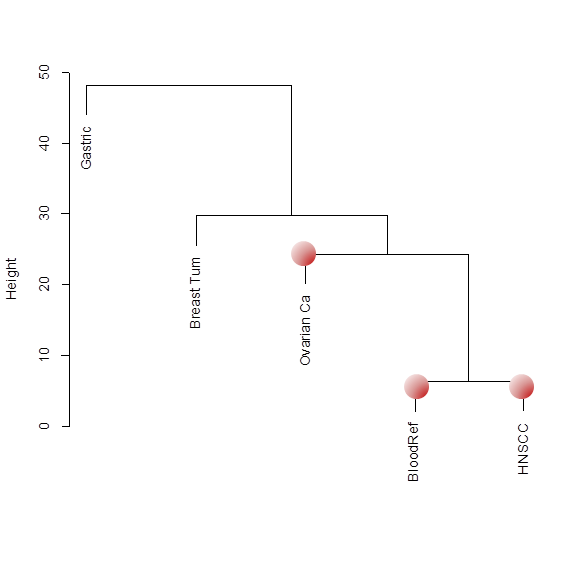 |
| --- | --- |
| C. First ten terms of expansion (**Δ**_10_)  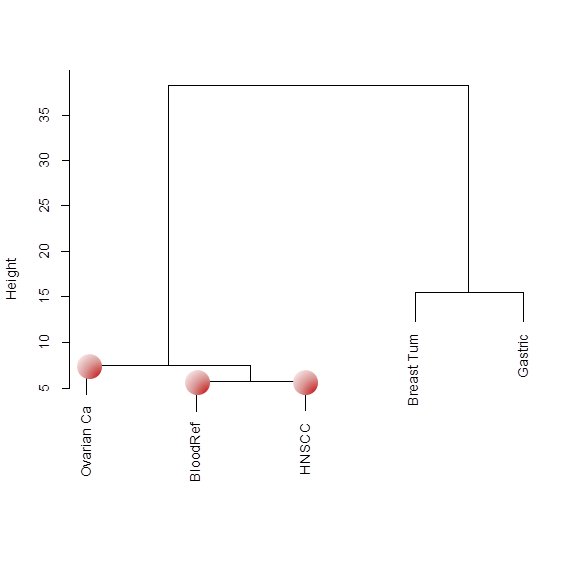 | D. Removing the first expansion term (**B**_1_)  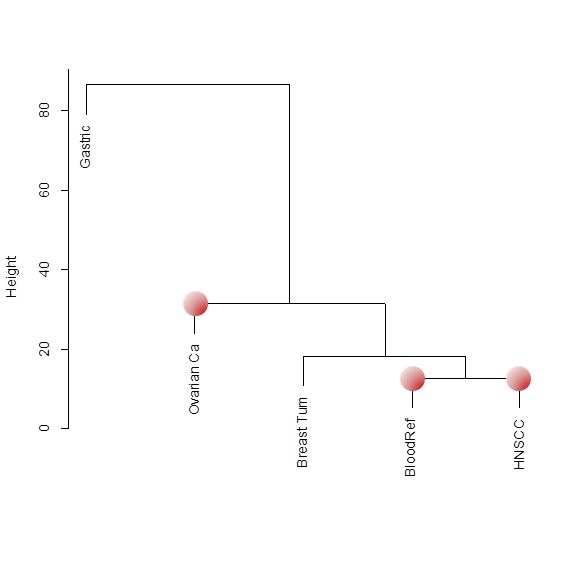 |

**Figure S20 - Correlation of Intercept coefficients with reference (whole blood) intercepts (27K)**

**Figure S21- Clustering of Δ and B intercept coefficients from 450K analyses**

| A. Total effect (**A**)  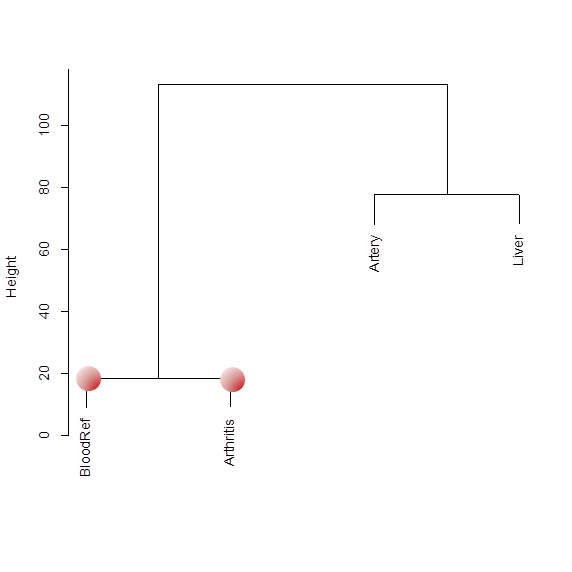 | B. First two terms of expansion (**Δ**_2_)  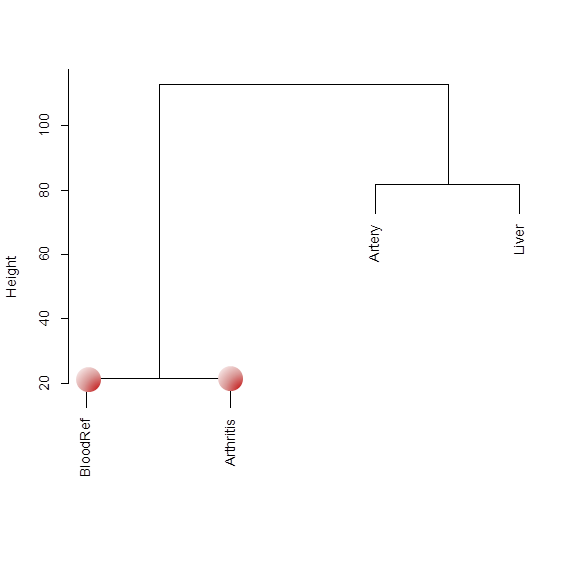 |
| --- | --- |
| C. First ten terms of expansion (**Δ**_10_)  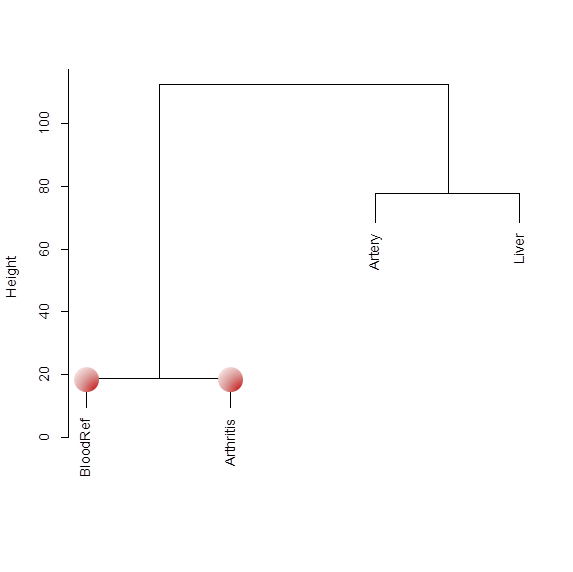 | D. Removing the first two expansion terms (**B**_2_)  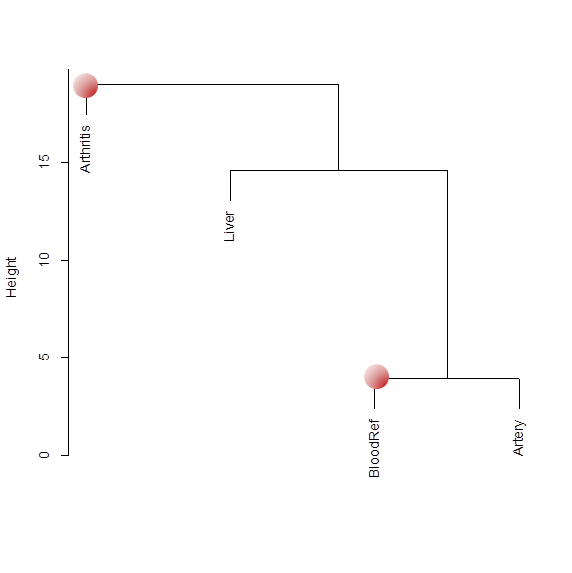 |

**Figure S22 – Correlation of Intercept coefficients with reference (whole blood) intercepts (450K)**

| A. Correlation of **Δ** intercepts with 450K blood reference **Δ** intercept | B. Correlation of **B** intercepts with 450K blood reference **B** intercept |
| --- | --- |

**Table S1 – Genes mapped to significant loci (q < 0.05 or top 50)**

**Source Code for Partitioning**

# Y = methylation matrix (numCpGs x numSubjects)

# xModel = covariate design matrix (numSubjects x numCovariates)

# MAXDIM = maximum value of k to try

# NBOOT = number of bootstraps to use for SE estimation

# OUTFILE = R workspace file name to which to save results

source("rfPartition.R")

# Gather compute-intensive quantities

theRF <- rfSlice(Y, xModel)

theSVD <- rfSVD(theRF$Bstar, theRF$Estar)

thePartition <- rfPartition2(theSVD, 1:dim(xModel)[2], MAXDIM)

partitionBootMoments <- array(0, dim=c(dim(thePartition), 2))

for(r in 1:NBOOT){

bootSV <- try( rfBootSVD(theRF) )

while(inherits(bootSV,"try-error")) bootSV <- try( rfBootSVD(theRF) )

bootPart <- rfPartition(bootSV, 1:dim(xModel)[2], MAXDIM)

# Running sum (for mean)

partitionBootMoments[,,,,1] <- partitionBootMoments[,,,,1] + bootPart

# Running sum of squares (for var)

partitionBootMoments[,,,,2] <- partitionBootMoments[,,,,2] + bootPart*bootPart cat(r,"\n")

if(r>3){

bootPartMean <- partitionBootMoments[,,,,1]/r

bootPartSqMean <- partitionBootMoments[,,,,2]/r

partitionVar <- bootPartSqMean-bootPartMean*bootPartMean

partitionVar[partitionVar<0] <- 0

partitionSD <- sqrt(partitionVar)

partitionTvalue <- thePartition/partitionSD

}

}

bootPartMean <- partitionBootMoments[,,,,1]/NBOOT

bootPartSqMean <- partitionBootMoments[,,,,2]/NBOOT

partitionVar <- bootPartSqMean-bootPartMean*bootPartMean

partitionVar[partitionVar<0] <- 0

partitionSD <- sqrt(partitionVar)

partitionTvalue <- thePartition/partitionSD

# Save compute-intensive results

save(list=c("thePartition","partitionSD"),file=OUTFILE)

# P-values and Q-values

partitionPvalue <- 2*pnorm(-abs(partitionTvalue)) # P-values for individual coefficients (not for contrasts)

partitionQvalObjectsB <- apply(partitionPvalue[,,1],2,safeQvalue)

partitionQvalObjectsD <- apply(partitionPvalue[,,2],2,safeQvalue)

# Various objective functions

diffDelta <- apply(abs(thePartition[,-1,,2,drop=FALSE]),1:2,diff)

diffDeltaSq <- apply(diffDelta*diffDelta,1:2,sum)

diffDeltaSqMed <- apply(diffDeltaSq,1,median)

diffTDelta <- apply(abs(partitionTvalue[,-1,,2,drop=FALSE]),1:2,diff)

diffTDeltaSq <- apply(diffTDelta*diffTDelta,1:2,sum)

diffTDeltaSqMed <- apply(diffTDeltaSq,1,median)

# Choose best value of k

kChooseEst <- which.min(diffDeltaSqMed)

kChooseTval <- which.min(diffTDeltaSqMed)

###################### CONTENTS OF rfPartition.R ###############################

library(limma)

library(qvalue)

rfSlice <- function(Y, X){

Bstar <- lmFit(Y,X)$coef

muStar <- Bstar %*% t(X)

Estar <- Y - muStar

muStar <- ifelse(muStar < 1e-05, 1e-05, muStar)

muStar <- ifelse(muStar > 0.99999, 0.99999, muStar)

dispersion <- sqrt(muStar * (1 - muStar))

Q <- Estar/dispersion

list(Bstar=Bstar, dispersion=dispersion, Estar=Estar, X=X, Q=Q)

}

rfSVD <- function(Bstar, Estar){

Estar[is.na(Estar)] <- 0

svd(cbind(Bstar,Estar))

}

rfBootSVD <- function(rf0){

n2 <- dim(rf0$X)[1]

iboot <- sample(1:n2, n2, replace = TRUE)

mu <- rf0$Bstar %*% t(rf0$X)

Yboot <- mu + rf0$dispersion * rf0$Q[, iboot]

BstarBoot <- lmFit(Yboot,rf0$X)$coef

EstarBoot <- Yboot - BstarBoot %*% t(rf0$X)

rfSVD(BstarBoot,EstarBoot)

}

rfPartition <- function(sv, coefs, d){

p <- length(coefs)

U <- sv$d*t(sv$v[coefs,,drop=FALSE])

B <- array(0, dim=c(dim(sv$u)[1], p, d, 2))

n <- length(sv$d)

for(i in 1:d){

B[,,i,1] <- sv$u[,i:n,drop=FALSE]%*%U[i:n,,drop=FALSE]

B[,,i,2] <- sv$u[,1:i,drop=FALSE]%*%U[1:i,,drop=FALSE]

}

B

}

safeQvalue <- function(p,...){

qv <- try( qvalue(p,...) )

if(inherits(qv,"try-error")){

qv <- list(qvalue=rep(1,length(p)), pi0=1)

}

if(length(qv)==1){

qv <- list(qvalue=rep(1,length(p)), pi0=1)

}

qv

}
